# Arsenic biosorption mediated by arsenic-binding proteins QueF and QueE in *Lysinibacillus* sp. OR-15

**DOI:** 10.1128/aem.00441-25

**Published:** 2025-04-25

**Authors:** Qing Xu, Weishi Tian, Hongliang Liu, Gejiao Wang, Kaixiang Shi

**Affiliations:** 1 National Key Laboratory of Agricultural Microbiology, College of Life Science and Technology, Huazhong Agricultural University 47895, Wuhan, China; 2 School of Life Sciences and Medicine, Shandong University of Technology 91620, Zibo, Shandong, China; Shanghai Jiao Tong University, Shanghai, China

**Keywords:** *Lysinibacillus*, arsenic resistance, arsenite biosorption, arsenite binding, transcriptional regulation

## Abstract

Microorganisms play a crucial role in arsenic transformation, with associated genes highly conserved within the *ars* operon. Although microorganisms can absorb arsenic, no specific genes responsible for intracellular sequestration have been found within this operon. This suggests that genes outside the *ars* operon may also contribute to bacterial arsenic resistance. Here, we identified an arsenic-resistant *Lysinibacillus* sp. OR-15 that exhibits consistent biosorption capacity for arsenic. Our findings indicate that the expression of heavy metal-related genes *queF* and *queE*, which are not located in the *ars* operon, is also induced by arsenite [As(III)]. When *queF* or *queE* is expressed in the arsenic-sensitive bacterium AW3110, it enhances both the resistance to As(III) and the biosorption capacity. Purified QueF and QueE demonstrate binding abilities for both As(III) and arsenate [As(V)]. Site-directed mutagenesis studies reveal that the conserved cysteine residue at position 101 in QueF and position 37 in QueE are critical for As(III) and As(V) binding. The transcriptional regulation mechanism involves the arsenic-responsive protein ArsR, which binds to the promoter region of the *que* operon and regulates its expression. This study elucidates the molecular mechanisms underlying QueF/QueE-mediated arsenic biosorption in *Lysinibacillus* sp. OR-15.

**IMPORTANCE:**

Arsenic is a ubiquitous metalloid pollutant in the environment, and its bioavailable concentration significantly influences its toxicity. Microorganisms play a crucial role in the geochemical cycling of arsenic, with certain species capable of reducing its bioavailability through biosorption. Consequently, elucidating the mechanisms of bacterial biosorption of As(III) is essential. This study identifies arsenic-binding proteins, QueF and QueE, which are regulated by ArsR in *Lysinibacillus* sp. OR-15. These proteins can directly bind intracellular As(III), facilitating its biological fixation and mitigating the toxic effects of As(III) to cells. This discovery provides valuable insights into the microbial mechanisms of microbial arsenic biosorption.

## INTRODUCTION

Arsenic, a well-known toxic metalloid, is ubiquitously distributed throughout the Earth’s crust (1). In its natural state, it predominantly occurs in inorganic forms such as arsenate [As(V)] and arsenite [As(III)], as well as in organic arsenic compounds (2). The toxicity of arsenic varies based on its chemical form, with the order generally being trivalent organic arsenic compounds > As(III) > As(V) > pentavalent organic arsenic compounds (3). Arsenic pollution of the environment has increased significantly as a result of both natural processes, such as volcanic activity and mineral weathering, and man-made ones, such as mining, burning fossil fuels, and excessive use of pesticides containing arsenic (4, 5). Arsenic contamination in groundwater and soil constitutes a critical environmental and public health challenge in numerous countries, including China, Vietnam, the United States, and the Bengal Delta (6, 7). Human health is seriously endangered when exposed to arsenic through tainted crops or drinking water (8). Long-term exposure to high amounts of arsenic can raise the risk of carcinogenesis and seriously harm important organs, including the liver and kidneys (9, 10).

Arsenic contamination in drinking water has emerged as a progressively severe global issue, with hundreds of millions of individuals worldwide being exposed to arsenic on a daily basis (11, 12). Arsenic is considered one of the most important environmental contaminants and dangerous substances due to its acute and chronic negative effects, including carcinogenicity (13). The transformation and environmental mobility of arsenic species are significantly influenced by microorganisms (14). Through prolonged evolutionary processes, microorganisms have typically localized arsenic resistance genes within arsenic resistance operons (*ars* operon) (15). In most microbial genomes, these operons typically comprise an As(III) sensing regulator, As(V) reductase, and As(III) permease (16). In response to intracellular concentrations of As(III), the ArsR repressor protein controls the expression of these operons (17). One of the main detoxifying processes in As-resistant bacteria is the efflux of several types of arsenic (18). By oxidizing As(III) to the less poisonous As(V) via the As(III) oxidase AioBA, bacteria may also detoxify arsenic, which is another important microbial detoxification mechanism (19). The microbial oxidation of highly toxic As(III) to the less toxic As(V) is also recognized as an effective approach for arsenic remediation (20). Arsenic oxidation enhances the absorption of arsenic by iron oxides in contaminated soils and also facilitates its precipitation and immobilization in polluted water bodies through the addition of chemical reagents (20, 21).

In recent years, research has highlighted the biosorption capabilities of various microorganisms toward As. Notably, 60% of As(III) could be extracted from its culture media by the halophilic bacteria *Halomonas elongata* SEK2 without oxidizing it to As(V) (22). *Alishewanella agri* PMS5 exhibited an adsorption capacity of 0.12 mg/g for As(III) (23). *Exiguobacterium* sp. As-9 and *Bacillus hornekiae* As-14 showed accumulation rates exceeding 60% for both As(V) and As(III) (24). It has been established that functional groups of extracellular polymers in the cyanobacterium *Synechocystis* PCC6803, including NH, OH, C = O, and C = C, play a role in arsenic bioaccumulation and transformation (25). However, the mechanisms underlying intracellular arsenic bioaccumulation in bacteria require further investigation.

Current research mostly focuses on ars operons because of the remarkable conservation of arsenic resistance genes, which are frequently grouped within these operons. This method, however, might ignore some genes that are essential to the metabolism of bacterial arsenic but are not a component of the *ars* operon. In our previous research, we examined the cadmium immobilization mechanism of *Lysinibacillus* sp. OR-15 and found that the *queF* gene enhances bacterial resistance to cadmium (26). Subsequently, we purified the QueF protein to investigate its function in cadmium binding *in vitro*, using As(III) as a negative control. Surprisingly, we discovered that QueF also has the ability to bind As(III), leading us to hypothesize that certain genes outside the *ars* operon may play a role in bacterial arsenic metabolism.

In this study, we observed that *Lysinibacillus* sp. OR-15 demonstrated both resistances to As(III) and effective bioaccumulation capabilities. The gene cluster comprising *queF*, *queC*, *queD*, and *queE* in *Lysinibacillus* sp. OR-15 was co-transcribed and induced by As(III). Both QueF and QueE were found to enhance As(III) resistance and bioaccumulation capabilities of arsenic-sensitive bacteria AW3110 by binding to As(III). Additionally, this study confirmed the regulatory role of ArsR in the expression of the *que* operon. Our findings elucidate the mechanism of intracellular arsenic bioaccumulation in *Lysinibacillus* sp. OR-15, thereby deepening our understanding of bacterial arsenic detoxification processes.

## RESULTS

### Arsenic resistance, removal phenotype, and gene analysis of *Lysinibacillus* sp. OR-15

**Fig 1:**
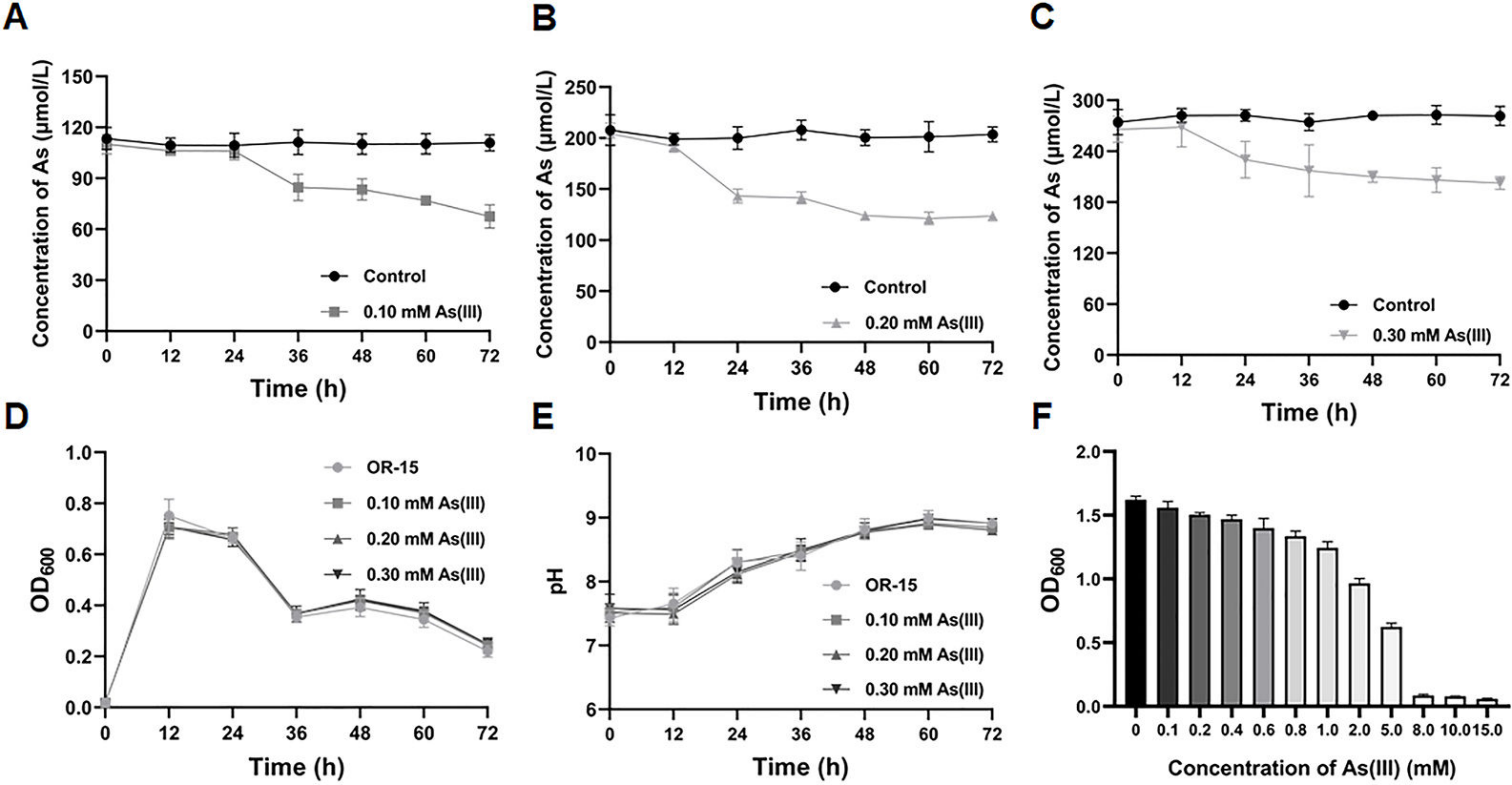
As(III) removal curve and resistance of *Lysinibacillus* sp. OR-15. (**A**) Removal curve of 0.10 mM As(III). (**B**) Removal curve of 0.20 mM As(III). (**C**) Removal curve of 0.30 mM As(III). (**D**) Growth curves at varying concentrations of As(III). (**E**) pH changes at different As(III) concentrations. (**F**) Determination of the maximum inhibitory concentration of As(III). These data represent average values obtained from three biological replicates. OD_600_, optical density at 600 nm.

The tolerance and accumulation capability of *Lysinibacillus* sp. OR-15 toward As(III) were evaluated in R2A medium. Under conditions of 0.10, 0.20, and 0.30 mM As(III), strain OR-15 specifically eliminated 36.24%, 29.54%, and 24.71% of total arsenic from the culture supernatant (Fig. 1A through C). The total amount of arsenic removed by bacterial strain OR-15 in the culture supernatant rose from 36.24 to 59.08 and 74.13 µM as the concentration of arsenic increased. This is associated with the higher intracellular arsenic concentration in bacteria cultured under high arsenic concentrations (Fig. 1A through C). No significant differences in bacterial growth, lag phase, and lysis phase were observed across varying As(III) concentrations (Fig. 1D). Furthermore, it was discovered that strain OR-15 raised the pH of the culture supernatant irrespective of the As(III) concentration (Fig. 1E). We utilized a pressure disruption device to treat bacterial cultures at various time points. Our results demonstrated that cell disruption raised the pH, indicating that bacterial lysis causes the culture medium’s pH to rise (Fig. S1). The maximum inhibitory concentration (MIC) of As(III) for strain OR-15 was determined to be 5 mM in R2A medium (Fig. 1F).

**Fig 2:**
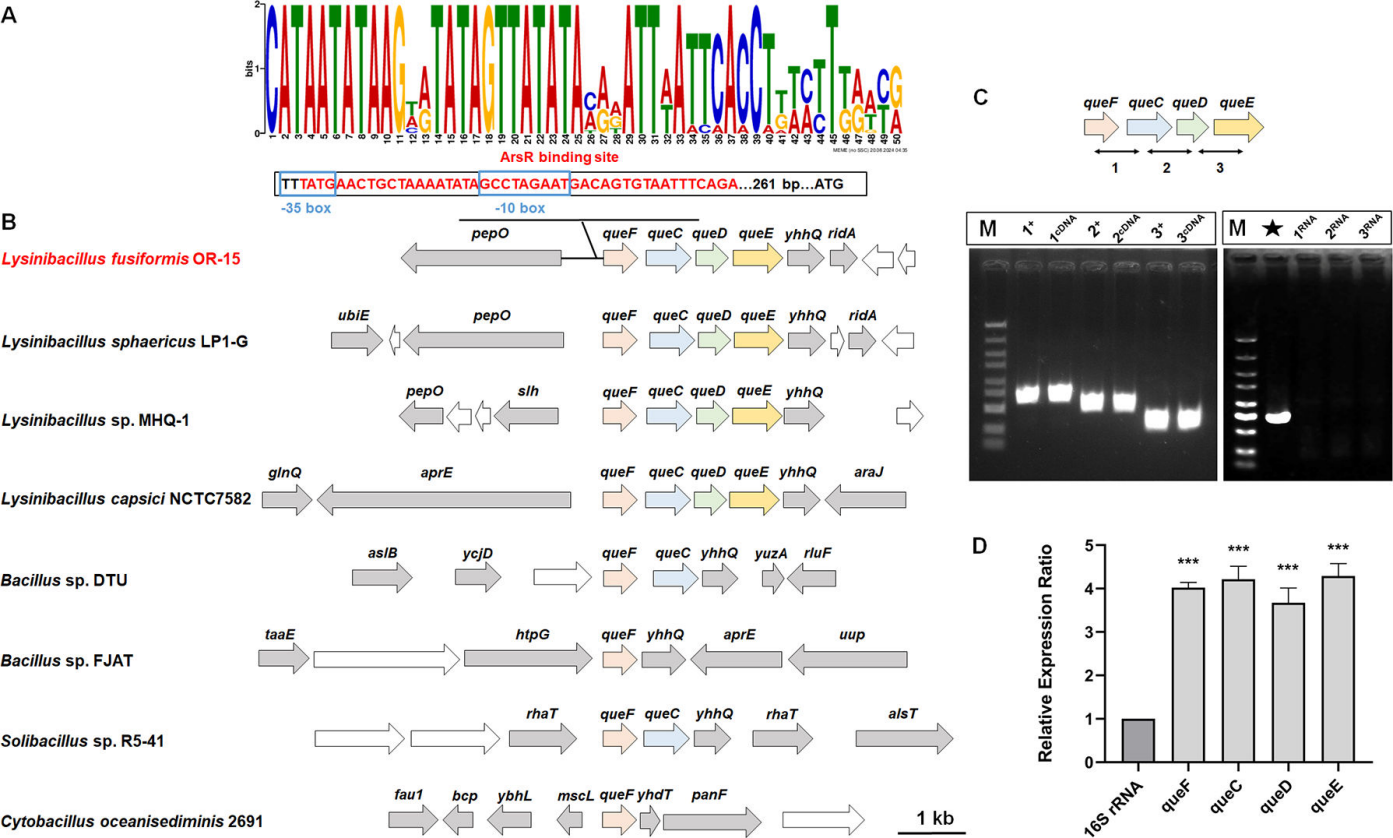
Analysis of *que* gene clusters. (**A**) The predicted ArsR binding motif within the que operon of *Lysinibacillus* sp. OR-15. (**B**) Comparative analysis of the gene arrangement of *que* gene clusters across various bacterial genomes. The accession numbers of these genomes are as follows: JAEIJF010000031.1 (*Lysinibacillus fusiformis* OR-15), CP071739.1 (*Lysinibacillus sphaericus* LP1-G), CP110804.1 (*Lysinibacillus* sp. MHQ-1), NZ_UAQE01000001.1 (*Lysinibacillus capsica* NCTC7582), CP135435.1 (*Bacillus* sp. DTU), CP012602.1 (*Bacillus* sp. FJAT), CP024123.1 (*Solibacillus* sp. R5-41), and NZ_CP015506.1 (*Cytobacillus oceanisediminis* 2691). (**C**) Co-transcriptional analysis of *queF*, *queC*, *queD,* and *queE*. M, molecular weight marker (GN5K). Markers 1, 2, and 3 represent common fragments shared among *queF*, *queC*, *queD,* and *queE*. The “+” marker means the use of total DNA as the template. The “cDNA” marker means the use of cDNA as the template. In lane ★, the 16S rRNA gene fragments were amplified using total DNA as the template. The “RNA” marker means the use of total RNA as the template. (**D**) Relative expression ratios of *queF*, *queC*, *queD,* and *queE* under As(III) stress compared with gene expression without As(III) as determined by reverse transcription-quantitative PCR. Data are presented as means of three biological replicates, with *** indicating a significant difference at a *P* value of <0.001.

Gene analysis of *Lysinibacillus* strains revealed that the genes *queF*, *queC*, *queD*, and *queE* are clustered together (Fig. 2A). We designed specific primers to amplify intergenic regions between these adjacent genes (see Table 2). Using cDNA as a template, we found that each pair of genes could be amplified into fragments identical to those obtained from genomic DNA templates (Fig. 2B). PCR results indicated that these genes are co-transcribed and located on the same transcript. Upon exposure to 0.1 mM As(III), the expression levels of *queF*, *queC*, *queD*, and *queE* in *Lysinibacillus* sp. OR-15 were upregulated, suggesting that this operon may play a role in As-related metabolic processes (Fig. 2C).

### QueF and QueE enhance bacterial resistance to As(III) and exhibit binding affinity to As(III)

**Fig 3:**
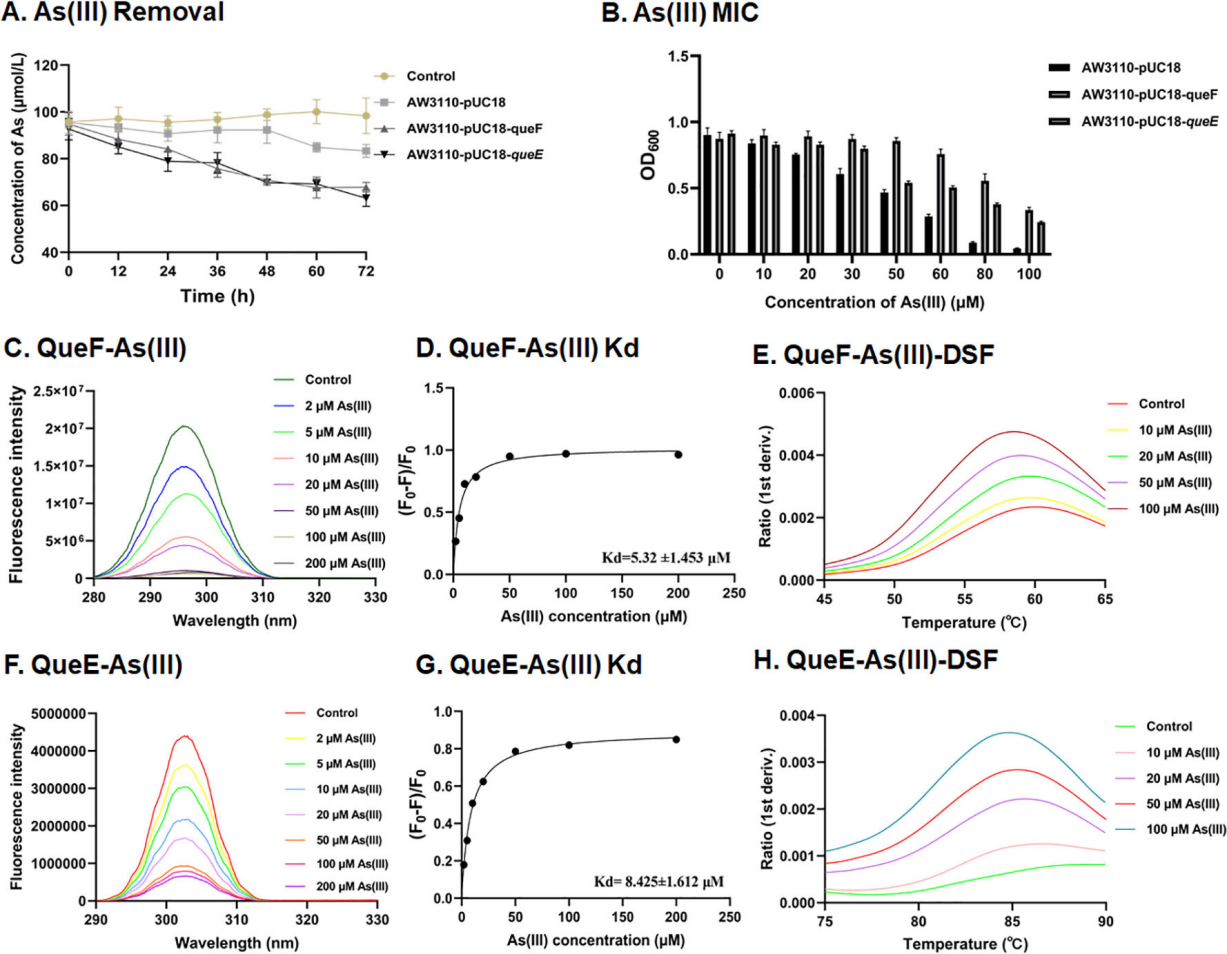
Phenotypic analysis of heterologous expression strains in As(III) removal, MIC, and binding affinity. (**A**) The As(III) removal curves of the *queF* and *queE* heterologous expression strain AW3110 in a medium containing 0.1 mM As(III). (**B**) Determination of the MIC of As(III) for the *queF* and *queE* heterologous expression strains. Data are presented as means of three biological replicates. (**C**) Fluorescence quenching was observed upon incubating purified QueF protein with varying concentrations of As(III) (0, 2, 5, 10, 20, 50, 100, and 200 mM). (**D**) The dissociation constant (Kd) for the binding of QueF to As(III) was calculated from fluorescence data analysis. (**E**) Binding interactions between QueF protein and As(III) at concentrations of 0, 10, 20, 50, and 100 mM were examined using nanoDSF. (F) Fluorescence quenching was observed when purified QueE was incubated with different concentrations of As(III) (0, 2, 5, 10, 20, 50, 100, and 200 µM). (G) The Kd value for the binding of QueE to As(III) was calculated based on fluorescence data. (H) Binding of QueE protein to As(III) at concentrations of 0, 10, 20, 50, and 100 µM was assessed by nanoDSF.

To investigate the functions of these four genes in As-related metabolic processes, we conducted heterologous overexpression experiments for each gene in the arsenic-sensitive *Escherichia coli* strain AW3110. The heterologous overexpression of the *queF* or *queE* gene in strain AW3110 enhanced the removal of total As from the culture supernatant (Fig. 3A), whereas overexpression of the *queC* or *queD* genes did not substantially improve As bioaccumulation of strain AW3110 (Fig. S2). Strain AW3110 with overexpressed *queF* or *queE* gene exhibited increased tolerance to As(III) (Fig. 3B). These results suggest that *queF* and *queE* genes play a crucial role in enhancing bacterial resistance to As(III). Therefore, we further investigated these two proteins in subsequent experiments.

We purified the QueF and QueE proteins *in vitro* and evaluated their binding abilities to As(III) using intrinsic tryptophan quenching and nano-differential scanning fluorimetry (nanoDSF). The fluorescence intensity of QueF exhibited a concentration-dependent quenching effect as the As(III) concentration increased (Fig. 3C). The dissociation constant (Kd) for the interaction between QueF and As(III) was determined to be 5.32 ± 1.453 µM (Fig. 3D). In nanoDSF assays, both the intrinsic fluorescence of the protein and the melting temperature showed consistent changes with increasing As(III) concentration compared to the untreated control (Fig. 3E). The purified QueE protein exhibited a concentration-dependent quenching of tryptophan fluorescence intensity with increasing As(III) concentration (Fig. 3F). The binding affinity of QueE to As(III), as determined by the Kd value, was 8.425 ± 1.612 µM (Fig. 3G). This interaction was further confirmed using nanoDSF analysis (Fig. 3H). Additionally, tryptophan fluorescence quenching experiments demonstrated that both QueF and QueE bind As(V), with respective Kd values of 5.240 ± 1.11 µM and 8.351 ± 3.051 µM (Fig. S3). The nanoDSF results also further supported the ability of QueF or QueE to bind As(V) (Fig. S3). However, neither QueF nor QueE exhibited binding affinity for Sb(III) (Fig. S4).

**Fig 4:**
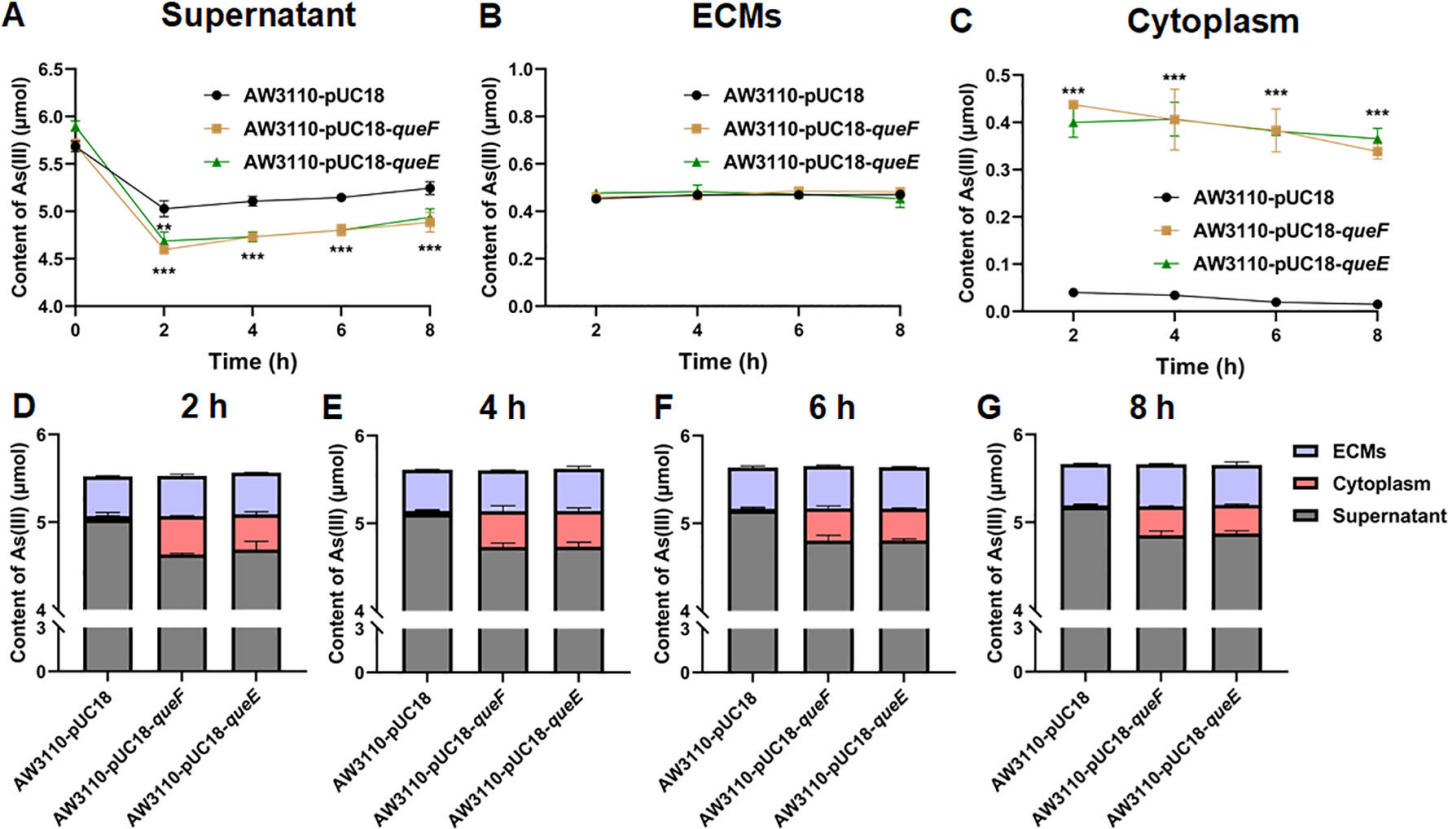
Subcellular content analysis of arsenic in heterologous expression strains. (**A–C**) Arsenic concentrations in the culture supernatant, extracellular matrix, and cytoplasm were measured across different treatment groups incubated for 2, 4, 6, and 8 h incubation. (**D–G**) Distribution of arsenic within various bacterial compartments at 2, 4, 6, and 8 h of incubation. ECM, extracellular matrix. Data are presented as means of three biological replicates, with ** indicating a significant difference at *P* < 0.01 and *** indicating a significant difference at *P* < 0.001.

Bioinformatics analysis of the transmembrane domains of QueF and QueE revealed that neither protein contains a transmembrane domain, indicating that they are classified as cytoplasmic proteins (Fig. S5). To further elucidate the role of QueF and QueE in arsenic bioremediation, we conducted fractionation experiments using arsenic-sensitive bacterial strains heterologously expressing these proteins. The results demonstrated that, compared to the control group, bacterial strains expressing QueF and QueE exhibited a significant decrease in arsenic levels in the culture supernatant (Fig. 4A), while no significant differences were observed in the arsenic content adsorbed by extracellular matrix (Fig. 4B). Conversely, a significant increase in intracellular arsenic was detected in these strains (Fig. 4C). In strains expressing QueF and QueE, there were notable increases in intracellular arsenic at 2, 4, 6, and 8 h (Fig. 4D through G), indicating that arsenic accumulation primarily occurred in the cytoplasm. Additionally, the total arsenic content in bacterial cells, measured at 2, 4, 6, and 8 h, correlated with the sum of arsenic detected in the fractionated cellular components (Fig. S6). Collectively, these findings suggest that QueF and QueE enhance bacterial arsenic bioremediation through intracellular arsenic binding.

### The conserved cysteine residues in QueF and QueE play a crucial role in As(III) binding

**Fig 5:**
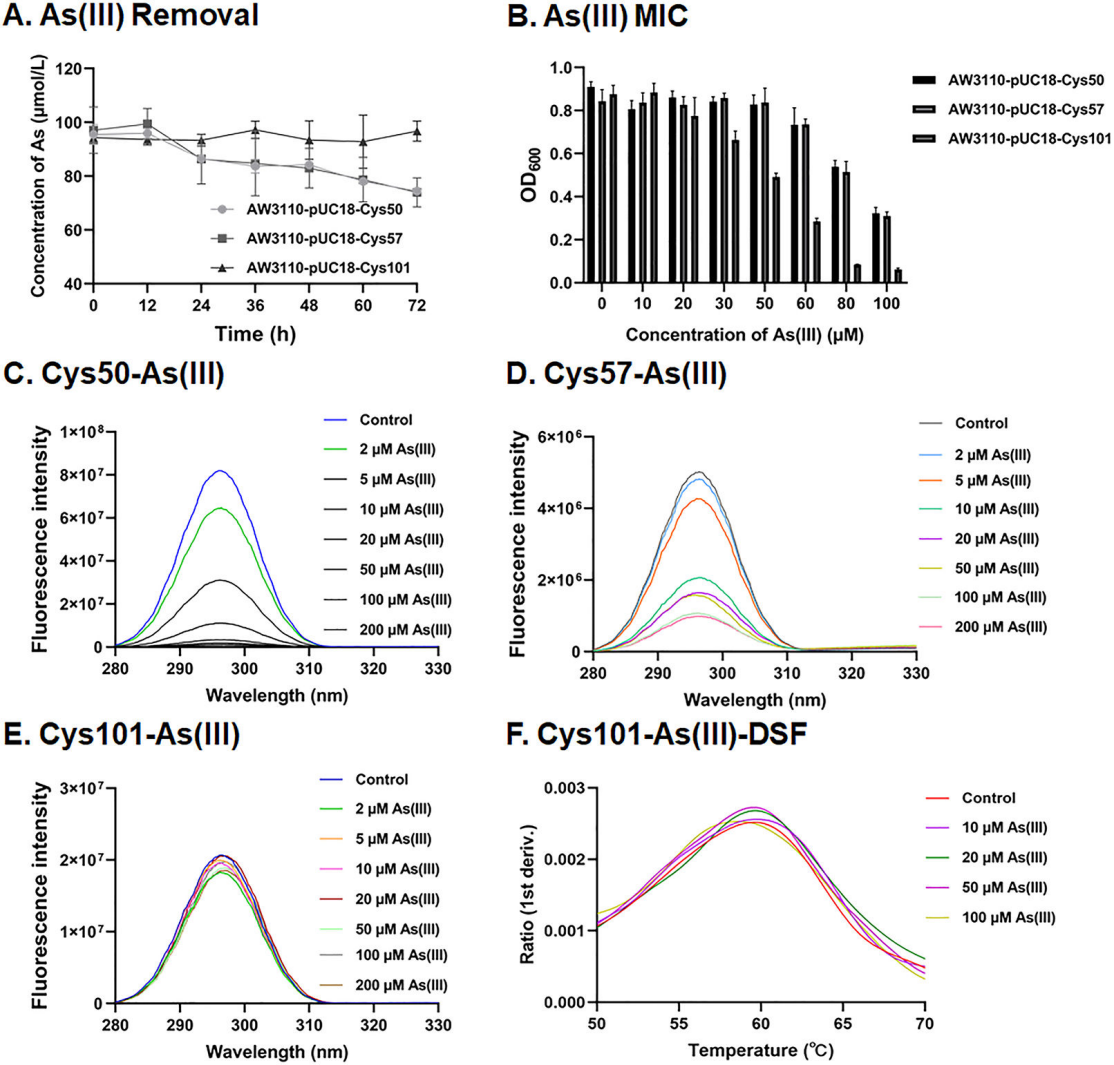
Phenotypic analysis of point mutant protein QueF in As(III) removal, MIC, and binding affinity. (**A**) The curve of As(III) removal by the strains heterologously expressing point mutant protein QueF strains in a medium containing 0.1 mM As(III). (**B**) The MIC of As(III) for strains heterologously expressing point mutant QueF protein. Data are presented as means of three biological replicates. Fluorescence quenching was observed with Cys50 (**C**) and Cys57 (**D**) point mutant proteins upon incubation with varying concentrations of As(III) (0, 2, 5, 10, 20, 50, 100, and 200 µM). For the Cys101 point mutant protein, both intrinsic tryptophan quenching (**E**) and differential fluorescence scanning (**F**) were tested across different As(III) concentrations.

In the QueF orthologs, three cysteine residues (Cys50, Cys57, and Cys101) were conserved (Fig. S7). To investigate the impact of these cysteine residues on bacterial As(III) bioaccumulation and resistance, point mutations at Cys50, Cys57, or Cys101 were introduced into strain AW3110. These results demonstrated that mutation at Cys101 impaired As bioaccumulation and reduced As(III) resistance in strain AW3110 (Fig. 5A and B). In contrast, mutations at Cys50 or Cys57 did not influence As bioaccumulation or resistance in strain AW3110. Cys50, Cys57, and Cys101 point-mutated proteins were purified to investigate which conserved cysteine residue is involved in QueF binding to As(III). Consistent with the results from heterologous expression of Cys101 in strain AW3110, the Cys101 mutant protein exhibited no significant changes in tryptophan fluorescence intensity across different concentrations of As(III) (Fig. 5E) or As(V) (Fig. S8) and showed no significant alterations in nanoDSF results (Fig. 5F; Fig. S8). Additionally, the Cys101 mutant protein did not exhibit binding to Sb(III) (Fig. S8). In contrast, the Cys50 and Cys57 mutants retained their ability to bind both As(III) and As(V) (Fig. 5C and D; Fig. S9). These findings collectively indicate that the conserved Cys101 residue of QueF plays a critical role in binding to As(III) and As(V).

**Fig 6:**
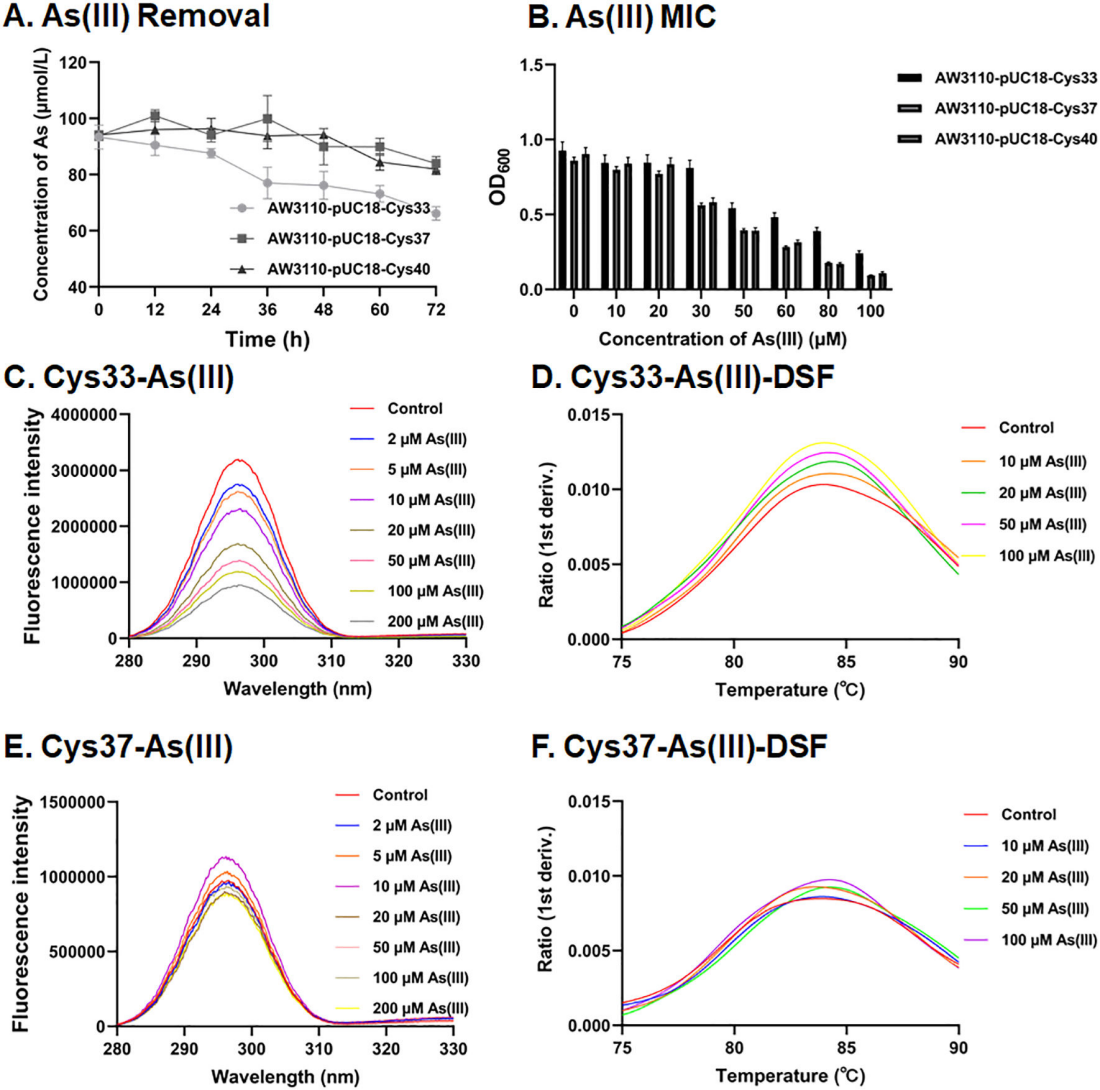
Phenotypic analysis of point mutant protein QueE in As(III) removal, MIC, and binding affinity. (**A**) The curve of As(III) removal by the strains heterologously expressing point mutant protein QueE strains in a medium containing 0.1 mM As(III). (**B**) The MIC of As(III) for strains heterologously expressing point mutant QueE protein. Data are presented as means of three biological replicates. Fluorescence quenching was observed with Cys33 (**C**) and Cys37 (**E**) point mutant proteins upon incubation with varying concentrations of As(III) (0, 2, 5, 10, 20, 50, 100, and 200 µM). Differential scanning fluorometry assays were conducted using the QueF mutant proteins Cys33 (**D**) and Cys37 (**F**).

The orthologs of QueE process conserved cysteine residues at positions Cys33, Cys37, and Cys40 (Fig. S10). Mutations at Cys37 and Cys40 reduced bioaccumulation and decreased resistance to As(III) in AW3110 (Fig. 6A and B). In contrast, mutation at Cys33 did not affect As(III) bioaccumulation or resistance in AW3110 (Fig. 6A and B). The QueE protein with a Cys37 mutation exhibited no gradient change in tryptophan fluorescence intensity across varying As(III) and As(V) concentrations, and nanoDSF results showed no consistent changes with increasing concentrations of As(III) and As(V) (Fig. 6E and F; Fig. S11). Mutation at Cys40 compromised the structural stability of QueE, leading to precipitation and aggregation of the purified protein. Conversely, the Cys33 mutant displayed a gradient change in tryptophan fluorescence intensity across different As(III) and As(V) concentrations, and nanoDSF results confirmed that the Cys33 mutation did not affect the binding affinity of QueE for As(III) and As(V) (Fig. 6C and D; Fig. S12). However, neither Cys37 mutant protein nor Cys33 mutant protein exhibited binding affinity for Sb(III) (Fig. S11 and S12). In summary, among the conserved cysteine residues in QueE, Cys37 plays a critical role in As(III) binding, whereas Cys40 contributes to the structural stability of QueE.

### ArsR regulates the expression of the *que* operon under As(III) conditions

**Fig 7:**
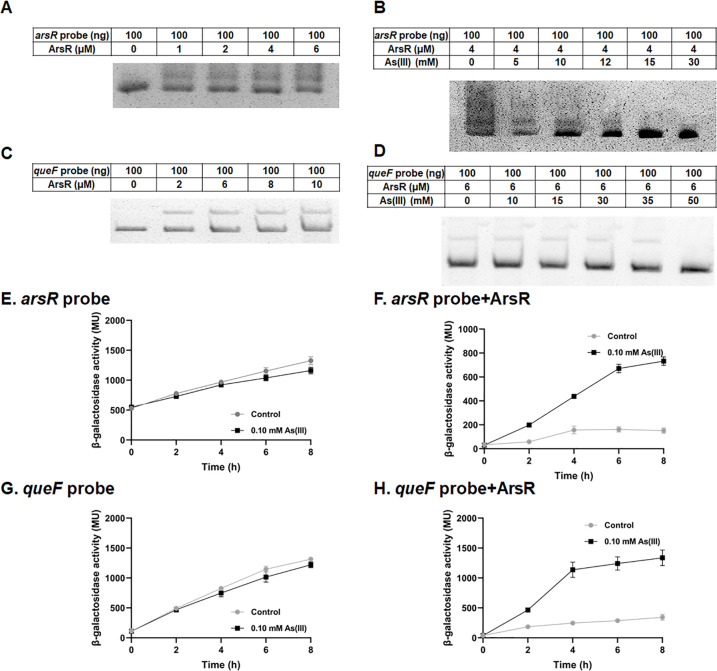
The regulation of *queF* under As(III) stress conditions by ArsR. The interaction between *arsR* probe (**A**) or *queF* probe (**C**) with ArsR protein was investigated. Competition EMSAs were conducted using *arsR* probe (**B**) or *queF* probe (**D**) and ArsR protein, along with increasing amounts of As(III). The amounts of DNA probes, ArsR protein, and As(III) are shown in the tables provided above each respective panel. The β-galactosidase activity was measured for strain DH5a carrying pLSP-*arsR* (**E**) or pLSP-*arsR*-ArsR (**F**) in LB medium supplemented with As(III). The β-galactosidase activity of strain DH5a harboring pLSP-*queF* (**G**) or pLSP-*queF*-ArsR (**H**) under identical conditions. Data are presented as means of three biological replicates.

To further investigate how the *que* operon in strain OR-15 senses As(III) signals, we conducted the *lacZ* reporter assay and electrophoretic mobility shift assay (EMSA). EMSA experiments were employed to investigate the binding ability of ArsR to the *que* promoter *in vitro*. Purified ArsR was incubated with either a 316 bp *arsR* regulatory sequence, which includes potential ArsR binding sites and serves as a positive control, or a 460 bp *que* regulatory sequence. The results demonstrated that ArsR caused shifts in the electrophoretic mobility of both the *arsR* and *que* promoter DNA (Fig. 7A and C). As the concentration of As(III) increased, the electrophoretic mobility shifts induced by ArsR gradually diminished for both the *arsR* and *que* promoter DNA (Fig. 7B and D). These findings confirm that ArsR can interact with the *que* regulatory region *in vitro* and that this interaction is attenuated in the presence of As(III). The results of the *lacZ* reporter assay demonstrated that expression from only the *arsR* promoter or the *que* promoter regions did not exhibit a significant correlation with As(III) levels (Fig. 7G and H). However, co-expression of the *arsR* promoter region with the ArsR encoding gene resulted in a significant enhancement of the reporter gene expression in response to As(III) (Fig. 7F). Similarly, co-expression of the *queF* promoter region with the ArsR encoding gene also led to a significant increase in reporter gene expression under As(III) exposure (Fig. 7H). The findings, corroborated by the results of the reporter gene assay and EMSA experiments, confirmed that ArsR regulated the *que* promoter in the presence of As(III) (Fig. 7).

## DISCUSSION

Arsenic, owing to its widespread industrial applications in semiconductors, catalysts, pigments, and various other products, has resulted in significant environmental accumulation (27). The cornerstone of heavy metal detoxification and remediation is not the elimination of the element but rather the reduction of its bioavailability, which in turn decreases cellular uptake of heavy metals. Microbial immobilization involves manipulating the speciation of arsenic to influence its bioavailability in soil, thus achieving effective arsenic immobilization (21). In recent years, microbial extraction techniques have garnered significant attention for remediating arsenic-contaminated water and soil (28). Bacteria can adsorb arsenic through extracellular polysaccharides on their cell wall surface or remove arsenic from the environment through intracellular accumulation (24, 25). Previous studies have demonstrated that the display of metal-binding protein on the bacterial surface to construct engineered bacteria holds potential for the remediation of heavy metal pollution (29, 30). Protein containing cysteine residues possesses the potential to bind As(III) due to its characteristic to form covalent As–S bonds with the thiol groups (31). However, the binding of arsenic to most proteins is detrimental to cellular activities. In this study, we discovered that the arsenic-regulated proteins QueF and QueE, which are induced by ArsR, serve as arsenic-binding proteins. This finding suggests that this mechanism may constitute an active and beneficial arsenic detoxification pathway in bacteria.

When microorganisms are exposed to arsenic in the environment, As(III) binds to the widely distributed arsenic operon regulator ArsR, thereby inducing the expression of ArsB and ArsC encoding genes (32, 33). ArsB facilitates the efflux of As(III) from the cell, while ArsC reduces As(V) to As(III), which is subsequently expelled by ArsB (32). Consequently, the predominant form of arsenic within bacterial cells is As(III), indicating that bacteria in arsenic-contaminated environments are continually subjected to oxidative stress induced by As(III). This study further demonstrates that ArsR regulates the expression of arsenic-binding proteins QueF and QueE encoding genes in *Lysinibacillus* sp. OR-15. The binding of arsenic by QueF and QueE reduces arsenic toxicity in a manner related to environmental arsenic concentrations. Given the widespread presence of ArsR-regulated As(III) efflux systems in bacteria, as environmental arsenic concentrations increase, the intracellular levels of free As(III) also rise. Bacteria can reduce cytotoxicity under high As(III) concentrations through the combined action of the ArsR-regulated arsenic efflux system and the intracellular arsenic-binding proteins QueF and QueE.

A novel bacterial arsenic detoxification mechanism has been identified in *Lysinibacillus* sp. OR-15. ArsR is capable of sensing intracellular As(III) signals and interacting with the upstream promoter region of the *que* operon. Upon cellular uptake of As(III), ArsR binds to As(III) and subsequently dissociates from the *que* operon promoter region, thereby initiating the expression of the *queF* and *queE* genes. The resultant proteins, QueF and QueE, then sequester intracellular free As(III), thereby reducing its toxicity by decreasing its bioavailability.

In this study, we have identified that arsenic-binding proteins QueF and QueE, which are regulated by ArsR, play a critical role in arsenic detoxification in bacteria. However, the genetic manipulation system of *Lysinibacillus* sp. OR-15 presents significant challenges for construction. The development of knockout or overexpression strains would facilitate a more comprehensive understanding of the specific roles of arsenic-binding proteins QueF and QueE in bacterial arsenic resistance. Currently, engineered strains featuring heavy metal-binding proteins on their surfaces have been developed for pollution remediation. The arsenic-binding proteins QueF and QueE identified in this study hold promise as potential biological materials for arsenic pollution remediation. However, further applications must rigorously evaluate the impact of protein assembly and stability in surface display on arsenic adsorption efficiency. Additionally, concerns regarding arsenic release from bacteria following the death of overexpressed engineered strains must be addressed.

## MATERIALS AND METHODS

### Strains and media

The strains used in this study, as detailed in Table 1, include the arsenic hypersensitive strain *Escherichia coli* AW3110 (Δ*ars*) (32, 34) and *Lysinibacillus* sp. OR-15 (26). These strains were cultured in lysogeny broth (LB) and R2A medium. The composition of 1 L of R2A medium is as follows: 0.5 g yeast exact, 0.5 g proteose peptone, 0.5 g casein hydrolysate, 0.5 g glucose, 0.5 g soluble starch, 0.3 g sodium pyruvate, 0.3 g dipotassium hydrogen phosphate, and 0.024 g magnesium sulfate. For 1 L of LB medium, the ingredients are 10 g NaCl, 10 g tryptone, and 5 g yeast extract.

**TABLE 1:** Strains and plasmid used in this research

Strain or plasmid	Description	Reference or source
Strains		
*Lysinibacillus* sp. OR-15		(26)
*E. coli* DH5α	supE44 lacU169(φ80lacZΔM15) hrdR17 recA1 endA1 gyrA96 thi-1 relA1	Invitrogen
*E. coli* BL21	F^−^ ompT hsdS_B_ (r_B_^−^ m_B_^−^) gal dcm me131 (DE3) pLysS (Cm^r^)	Invitrogen
*E. coli* AW3110	Δ*arsRBC*::cam F-IN(rrn-rrnE)	(34)
Plasmids		
pUC18	Amp^r^, clone and expression vector	MiaoLingBio
pLSP-KT2lacZ	Km^r^, oriV, lacZ-fusion vector used for *lacZ* fusion constructs	(15)
pET-28(a+)	Km^r^, His6 tag expression vector	Novagen
pET-32(a+)	Amp^r^, His6 tag expression vector	Novagen

### Plasmid construction

The genes coding for QueF (MBI6864646.1), QueC (MBI6864645.1), QueD (MBI6864644.1), and QueE (MBI6864643.1) were cloned from *Lysinibacillus* sp. OR-15 (accession number NZ_JAEIJF000000000) and expressed in the pUC18 plasmid, which were subsequently transformed into *E. coli* DH5α. The resulting recombinant plasmids were then transformed into *E. coli* AW3110 for heterologous expression experiments. For protein purification assays, the *queF* and *queE* genes were subcloned into the pET32a vector and transformed into *E. coli* BL21 for protein expression. The primers used for constructing these plasmids are shown in Table 2.

**TABLE 2:** Primers used in this research^*a*^

Primer	Sequence
Co-transcription assays	
*queF-queC*-F	TTATGCCCGCTTACCA
*queF-queC*-R	GTCCAGGTACAAATGTCGA
*queC-queD*-F	TACCTGGACGAAACCT
*queC-queD*-R	CTAAGTCCCAATACAACAC
*queD-queE*-F	ACGCTTCCACCTATGA
*queF-queC*-R	AAGATGCTTTCCGCTA
RT-qPCR assays	
*queF*-F	ATGCCCGCTTACCAATCAAC
*queF*-R	AACTTCCCCCAAACCTCGAT
*queC*-F	CGCTGGTGTTTTAGCGAGTC
*queC*-R	CAAACTGATCGGCAAGCTCC
*queD*-F	CAGATGAACGCGGCTTGATG
*queD*-R	CTCATCACGCAAGGCCACTA
*queE*-F	CGTCTTGGGGGTAATGGCTT
*queE*-R	CTAGGCGCCTTGGGAGAAAT
Heterologous expression assays	
*queF*-pUC18-F	AAAGGATCCTTGAAAGTGTTGGGAAAC
*queF*-pUC18-R	AAAAAGCTTAACGGTGTATCAATCACAAAGGA
*queC*-pUC18-F	AAAAGGATCCCATGACCTTTATCCAGAA
*queC*-pUC18-R	AAAAAAGCTTTTTACATTTGCCCTCA
*queD*-pUC18-F	AAAGGATCCTCGGGAAAGAACATTG
*queD*-pUC18-R	AAAAAGCTTCACCACGAACAAGAATAA
*queE*-pUC18-F	AAAAGGATCCCTATGCAGAAGCGAGAC
*queE*-pUC18-R	AAAAAGCTTCAATAAACAGCCCAAAC
Protein purification assays	
*queF*-pET32a-F	AAAGGATCCATTTTACAAGGAGGTTT
*queF*-pET32a-R	AAAAAGCTTCGCTCGATATCGATTATCA
*queC*-pET32a-F	AAAGGATCCGTGTAACTATGGGAAGCC
*queC*-pET32a-R	AAAAAGCTTTCTGACATGCTTGAAACTC
*queD*-pET32a-F	AAAGGATCCGGAAAGAACATTGACTTGCTA
*queD*-pET32a-R	AAAAAGCTTAACAGGTATCTAACTCAACTTCC
*queE*-pET32a-F	AAAGGATCCCTTATCGTGTTGTATTGGGACT
*queE*-pET32a-R	AAAAAGCTTACACTCTACACCTCTTTTATTTCC
Protein point mutation assays	
QueF	
*queF*-F	AAAGGATCCATTTTACAAGGAGGTTT
*queF*-R	AAAAAGCTTCGCTCGATATCGATTATCA
Cys50-F1	TCGTTAAGTTTAATGCACCAGAGTTCACTAGTTTA
Cys50-R1	AACTAGTGAACTCTGGTGTATTAAACTTAACGAAATAA
Cys57-F1	CCAGAGTTCACTAGTTTAGCACCGCTTACCAAT
Cys57-R1	CAGGTTGATTGGTAAGCGGTGCTAAACTAGTGAA
Cys101-F1	TGAAGATGCAGTTAACATCATTATGGAT
Cys101-R1	CCATAATGATGTTAACTGCATCTTCATGGA
QueE	
*queE*-F	AAAAGGATCCCTATGCAGAAGCGAGAC
*queE*-R	AAAAAGCTTCAATAAACAGCCCAAAC
Cys33-F1	GGCTGGCGCAGATTATTCTTGTTCG
Cys33-R1	GAATAATCTGCGCCAGCCGTTCTT
Cys37-F1	TGATTATTCTGCATCGTGGTGTGAT
Cys37-R1	CACACCACGATGCAGAATAATCACA
Cys40-F1	CGTGGGCAGATTCGTCGTTTACGT
Cys40-R1	ACGACGAATCTGCCCACGAACAAGAA
Electrophoretic mobility shift assays	
*queF*-promoter-F	GTTTGAAAATATGTTAGGAGCTT
*queF*-promoter-R	GAGGCTTTACCAATTCATCTA
*arsR*-promoter-F	AAAGCACAGTAACTAAGAAGC
*arsR*-promoter-R	CACCTCCACAAAATCACATAC
*arsR*-pET28a-F	AAAGGATCCTGCAATTTCCAATCCGTGTA
*arsR*-pET28a-R	AAAAAGCTTATACGCATGATCAGTCCCTCC
Reporter gene assays	
*queF*-promoter-F	AAAGAATTCGTTTGAAAATATGTTAGGAGCTT
*queF*-promoter-R	AAAGGATCCGAGGCTTTACCAATTCATCTA
*queF*-promoter-ArsR-F1	AAAGAATTCTGCGAAGATCATTTCCAACA
*queF*-promoter-ArsR-R1	TTCCATGAGGCTTTACCAATT
*queF*-promoter-ArsR-F2	ATTGGTAAAGCCTCATGGAAA
*queF*-Promoter-ArsR-R2	AAAGGATCCAACGAATATCCAAAGGGTAAG
*arsR*-promoter-F	AAAGAATTCAAAGCACAGTAACTAAGAAGC
*arsR*-promoter-R	AAAGGATCCCACCTCCACAAAATCACATAC
*arsR*-promoter-ArsR-F	AAAGAATTCTGCAATTTCCAATCCGTGTA
*arsR*-promoter-ArsR-R	AAAGGATCCTTACGCATGATCAGTCCCTCC

^*a*^ The underlined sequence denotes the restriction enzyme sites.

### Prediction of ArsR putative binding sites

The putative ArsR binding motif was predicted by MEME online software (http://meme-suite.org/tools/meme) (18). Using the putative ArsR binding motif, the ArsR binding sites in the genomes of various *Lysinibacillus* strains were predicted using the FIMO online software. The strains analyzed include *Lysinibacillus* sp. OR-15 (JAEIJF000000000.1), *L. sphaericus* G25-115 (GCA_015845635.1), *L. fusiformis* G25-113 (GCA_015845625.1), *L. sphaericus* OT4b.49 (GCA_001623495.1), *L. sphaericus* Gar NS 2 (GCA_035792175.1), *L. fusiformis* MGMM7 (CP130331.1), *L. capsici* TSBLM (CP122283.1), *L. fusiformis* TZA38 (CP141829.1), *L. pakistanensis* LY1 (CP126101.1), and *L. capsica* FN9 (CP106862.1). The prediction was performed with a match value parameter set at 0.0001.

### Co-transcription assays and reverse transcription-quantitative PCR (RT-qPCR)

Strain OR-15 was harvested by centrifugation at 12,000 rpm for 2 min after being cultured in a 100 mL flask of R2A medium for 24 h at 28℃ and 150 rpm. Total RNA extraction was performed using the Trizol method. Residual DNA was removed, and the RNA was reverse-transcribed into cDNA using HiScript II Q RT SuperMix for qPCR (+gDNA wiper) (Vazyme Biotech, R223). The cDNA served as the template for PCR amplification. To test the co-transcription of *queF* and *queC* genes, the primer pairs *queF-queC*-F/*queF-queC*-R were utilized (Table 2). Similarly, the primer pairs *queC-queD*-F/*queC-queD*-R and *queD-queE*-F/*queD-queE*-R were used to examine the co-transcription of *queC* and *queD* genes and *queD* and *queE* genes, respectively (Table 2). The primers *queF-queC*-F and *queF-queC*-R were used for testing the co-transcription of *queF* and *queC* genes, and the annealing temperature is 50°C. The annealing temperatures of primers *queF-queC*, *queC-queD*, and *queD-queE* were 50°C, 44°C, and 45°C. All PCR program extension times were 15 s. PCR products were analyzed by electrophoresis on a 1% agarose gel. The total DNA from strain OR-15 served as the positive control, while RNA served as the negative control and cDNA as the experimental sample.

For the RT-qPCR analysis, OR-15 cells were inoculated in R2A medium broth for 36 h, either supplemented with or devoid of 0.1 mmol/L As(III), under conditions of 28°C and 150 rpm. Cells were harvested by centrifuging at 12,000 rpm for 2 min. Subsequently, cDNA was synthesized according to the previously described protocol. The RT-qRCR PCR reaction mix was AceQ qPCR SYBR Green Master Mix (Low ROX Premixed) (Vazyme Biotech, Q131 version 22.1). The warm-up procedure of reverse transcription PCR was 95°C for 30 s. The PCR amplification consists of 40 cycles, with each cycle comprising denaturation at 95°C for 15 s, 60°C for 15 s, and 72°C for 30 s. The melting curve analysis is performed with 60°C for 60 s and 95°C for 15 s. Fluorescence signals were detected during both the amplification and melting curve stages. RT-qPCR assays were performed using the Lonza-Amaxa QUANTSTUDIO3 system, employing cDNA as the template. The specific primers utilized are detailed in Table 2, while the 16S rRNA gene served as the reference.

### Bacterial As(III) bioaccumulation and resistance assays

Bacterial As(III) bioaccumulation was investigated at various concentrations of As(III) (0.1, 0.2, and 0.3 mM) in R2A medium, with an inoculum size of 1% and incubation conditions of 28°C and 150 rpm. Culture samples were collected at 0, 12, 24, 36, 48, 60, and 72 h to determine strain growth (optical density at 600 nm [OD_600_]), pH changes, and As concentration. The As concentration was determined using high-performance liquid chromatography hydride generation atomic fluorescence spectroscopy (HPLC-HG-AFS) (AFS-SA-50; Beijing Jitian Instruments Co., Ltd., China) after centrifuging and filtering the culture supernatant (12,000 rpm for 2 min; 0.22 µm filter). Bacterial resistance to As(III) was evaluated based on strain growth (OD_600_) under different As concentrations. Strain OR-15 was inoculated into R2A medium containing As(III) concentrations ranging from 0 to 15.0 mM (in increments of 0.1, 0.2, 0.4, 0.6, 0.8, 1.0, 2.0, 5.0, 8.0, 10.0, and 15.0 mM) and cultivated for 48 h at 28°C and 150 rpm, followed by measurement of the absorbance of the bacterial culture at 600 nm.

### Heterologous expression assays

The plasmids used for heterologous expression experiments included pUC18-*queF*, pUC18-*queC*, pUC18-*queD*, and pUC18-*queE*. The recipient strain was AW3110, which was cultured in R2A medium at 37°C with 150 rpm. Strain AW3110 harboring the empty pUC18 plasmid served as the control group. The bioaccumulation and resistance of As(III) in AW3110 strains heterologously expressing the *queF*, *queC*, *queD*, and *queE* genes were evaluated using the previously described same methods. Bioaccumulation was assessed at a concentration of 0.10 mM As(III), while As resistance was tested at concentrations of 0, 10, 20, 30, 50, 60, 80, and 100 µM As(III).

### Protein purification *in vitro*

The plasmids used for protein purification were pET32a-*queF*, pET32a-*queE*, and pET28a-*arsR*, with *E. coli* BL21 serving as the recipient strain (Table 1). Cultivation of strains was conducted in LB medium at 37°C and 150 rpm. Upon reaching an OD_600_ of 0.3, induction was initiated by adding isopropyl β-D-thiogalactoside to a final concentration of 0.1 mM. The cultures were then incubated at 16°C for 20 h. Cells were harvested by centrifugation at 8,000 rpm for 8 min at 4°C and resuspended in binding buffer composed of 0.2 M borate saline (pH 8.4), 2.5 M NaCl, and 5 mM imidazole. Bacterial cells were lysed by a low-temperature ultra-high-pressure continuous flow cytometer (JN-MiniPro) and subsequently subjected to centrifugation at 8,000 rpm for 20 min at 4°C. The supernatant was incubated with 2 mL of Ni-NTA His-Bind resin (Smart-Lifesciences) and gently agitated at 4°C for 1 h. The proteins were subsequently eluted from resin using 10 mL of elution buffer, which consisted of 0.2 M borate saline (pH 8.4), 2.5 M NaCl, and 100 mM imidazole. To remove imidazole and ions, the protein eluate was dialyzed against ultrapure water on ice for 8 h. Finally, the protein solution was concentrated via ultrafiltration using a 10 kDa cutoff filter. The purity of the protein was assessed by SDS-PAGE, and its concentration was determined spectrophotometrically using a NanoDrop 2000 instrument (Thermo Fisher Scientific).

### Fractionation of cellular components for arsenic removal in heterologous expression strains

The plasmids used for intracellular protein adsorption experiment were pUC18-*queF* and pUC18-*queE*, and the recipient strain was AW3110. AW3110 strain was cultured in 100 mL R2A medium for 24 h (37°C, 150 rpm). Strain AW3110 containing pUC18 was the control group. The culture was collected by centrifugation (5,000 × *g*, 10 min) and resuspended with 0.85% NaCl containing 1 mM As(III). Strains were incubated statically for 2, 4, 6, and 8 h at 37°C. After different time exposures to As(III), the bacterial culture was divided into two identical parts, and cells were collected separately by centrifugation (5,000 × *g*, 10 min). Each experimental group was accompanied by three parallel groups, and the biomass of the strains was essentially uniform among each parallel group. One portion was used for the determination of total arsenic content in the bacterial cells, while the other part was used for the measurement of arsenic content in different fractions of the bacteria. The total arsenic content in the bacterial precipitate was determined by digestion using a mixture of nitric acid and perchloric acid (9:1). The methods for determining the arsenic content in different parts of the bacterial cells are as follows. The sediment of 5 mL simple was resuspended with 2 mL 1.5 M NaCl solution and centrifuged (5,000 × *g*, 10 min) to separate polymeric extracellular matrices (35). The bacterial precipitate was resuspended in 2 mL of 0.85% NaCl and lysed by a low-temperature ultra-high-pressure continuous flow cytometer (JN-MiniPro). Subsequently, the cytoplasm and the remaining components were separated by centrifugation (6,000 × *g*, 10 min). The arsenic content in the cytoplasm can be inferred by detecting the arsenic in the supernatant. The acid-digested precipitate can be used to determine the arsenic content in other cellular components. The concentration of arsenic in each component was tested by HPLC-HG-AFS (AFS-SA-50; Beijing Jitian Instruments Co., Ltd.).

### Tryptophan fluorescence quenching assays

To investigate the binding specificity of proteins with heavy metals, fluorescence assays were performed using a Spectro fluorophotometer (Beckman Coulter Tech, RF-5301PC). The excitation wavelength was set at 295 nm, while the emission wavelength ranged from 200 to 400 nm. Solutions of purified QueF or QueE proteins (1 µM) were mixed with varying concentrations of As(III), As(V), or Sb(III) (0, 2, 5, 10, 20, 50, 100, and 200 µM). Tryptophan fluorescence intensity was measured for each mixture. The maximum fluorescence values obtained at different concentrations of As(III), As(V), or Sb(III) were compared against the control group to determine the fluorescence quenching rate [formula: $\left(\frac{\left(F_{0}-F\right)}{F_{0}}\right)$].

### Differential scanning fluorometry assays (nanoDSF)

Real-time simultaneous monitoring of the intrinsic tryptophan fluorescence at 330 and 350 nm of protein was conducted using a Prometheus NT.48 instrument (Nano Temper Technologies) with an excitation wavelength of 285 nm. The purified QueF or QueE proteins were prepared at a concentration of 10 µM in ultrapure water for differential scanning fluorimetry measurement. To verify the effect of heavy metals on protein stability, purified proteins were incubated with varying concentrations of As(III), As(V), or Sb(III) (0, 10, 20, 50, and 100 µM). Samples (10 µL) were loaded into capillaries and placed in a sample holder. A temperature gradient ranging from 20°C to 90°C at a rate of 2°C/min was applied, and intrinsic protein fluorescence emissions at 330 and 350 nm were monitored (36).

### Protein point mutation

Mutants in the QueF and QueE protein were generated using the encoding gene site-directed mutagenesis, employing the primers listed in Table 2. Specifically, the residues Cys50, Cys57, and Cys101 in QueF were mutated to C50A, C57A, and C101A, respectively. Similarly, the residues Cys33, Cys37, and Cys40 in QueE were altered to C33A, C37A, and C40A. The point-mutated protein genes were subsequently cloned into the pUC18 plasmid and introduced into AW3110 to evaluate bacterial As(III) bioaccumulation and resistance. The pET32a expression vector harboring the point-mutated proteins was constructed in BL21 for subsequent protein purification. Assays for As(III) bioaccumulation and resistance, protein purification, and As(III) binding were performed as previously described. The primers are detailed in Table 2.

### EMSA

The DNA sequences utilized in this experiment comprised the promoter regions of the *queF* and *arsR* genes from the OR-15 genome. The amplification primers are detailed in Table 2. The protein employed for this assay was the ArsR protein. The reaction mixture was incubated at 28℃ for 30 min in a binding buffer consisting of 10 mM Tris−HCl (pH 7.5), 1 mM EDTA, 0.1 M KCl, 0.01 mg/L BSA, 5% glycerin, and 0.1 mM dithiothreitol. Following incubation, loading buffer consisting of 1 mM Tris−HCl (pH 7.5), 0.1 mM EDTA, and 5% glycerin was added to the reaction mixture, which was then subjected to native polyacrylamide gel electrophoresis on 6% acrylamide gels for protein separation. In the *arsR* promoter-ArsR experimental group, TGE buffer (12 mM Tris, 95 mM glycine, 0.5 mM EDTA, pH 8.0) was used for electrophoresis conducted at 80 V for 2 h. In contrast, in the *queF* promoter-ArsR group, electrophoresis was performed using TGE buffer (pH 10.24) at 80 V for 80 min. The gel was subsequently analyzed using a phosphor imaging system (iBright CL1000, Invitrogen) to investigate DNA-protein binding.

### Reporter gene assays

The plasmid used was pLSP-KT2lacZ, and the bacterial strain employed was DH5α (Table 1). Constructs were generated for the following elements: the promoter region of the *queF* gene, the *queF* gene promoter in conjunction with the ArsR protein encoding gene, the promoter region of the *arsR* gene, and the *arsR* gene promoter, in combination with the ArsR protein encoding gene. The primers used for these constructs are presented in Table 2. The constructed plasmids were subsequently transformed into DH5α cells. Bacterial cultures were inoculated into 5 mL of LB medium and incubated either in the presence or in the absence of 0.1 mM As(III) at 37°C and 150 rpm. Samples were collected at 0, 2, 4, 6, and 8 h post-inoculation to determine β-galactosidase activity in the culture supernatant (37).

### Statistical analysis

One-way analysis of variance was conducted via SPSS version 16.0 to assess significant differences among various treatments. Means and standard deviations were calculated with Microsoft Office Excel 2016. In cases where significant differences (*P* < 0.05 or *P* < 0.01) were identified, multiple comparisons were performed using the least significant difference test. The reliability of the experimental data was evaluated using Student’s *t*-test.

## SUPPLEMENTAL MATERIAL

Supplemental figures aem.00441-25-s0001.docx Figures S1 to S12.

ASM does not own the copyrights to Supplemental Material that may be linked to, or accessed through, an article. The authors have granted ASM a non-exclusive, world-wide license to publish the Supplemental Material files. Please contact the corresponding author directly for reuse.
