# Supplementary material for: Arsenic biosorption mediated by arsenic-binding proteins QueF and QueE in Lysinibacillus sp. OR-15
Source: Appl Environ Microbiol. 2025 Apr 25;91(5):e00441-25. doi: 10.1128/aem.00441-25 (PMC12093948; doi:10.1128/aem.00441-25)
Supplement: Supplemental figures — Figures S1 to S12. [file aem.00441-25-s0001.docx]

Supporting Information for

**Arsenic biosorption mediated by arsenic-binding proteins QueF and QueE in *Lysinibacillus* sp. OR-15.**

Qing Xu^a1^, Weishi Tian^a1^, Hongliang Liu^b^, Gejiao Wang^a^, Kaixiang Shi^a^*

a: National Key Laboratory of Agricultural Microbiology, College of Life Science and Technology, Huazhong Agricultural University, Wuhan, 430070, P. R. China

b: School of Life Sciences and Medicine, Shandong University of Technology, Zibo 255000, Shandong Province, People’s Republic of China

***** Corresponding author:

Kaixiang Shi, kaixiangshi@mail.hzau.edu.cn, National Key Laboratory of Agricultural Microbiology, Huazhong Agricultural University, Wuhan 430070, P. R. China.

^1^ Qing Xu and Weishi Tian contributed equally to this work. Author order was determined by decreasing seniority.

Number of Pages: 11

Number of Figures: 12


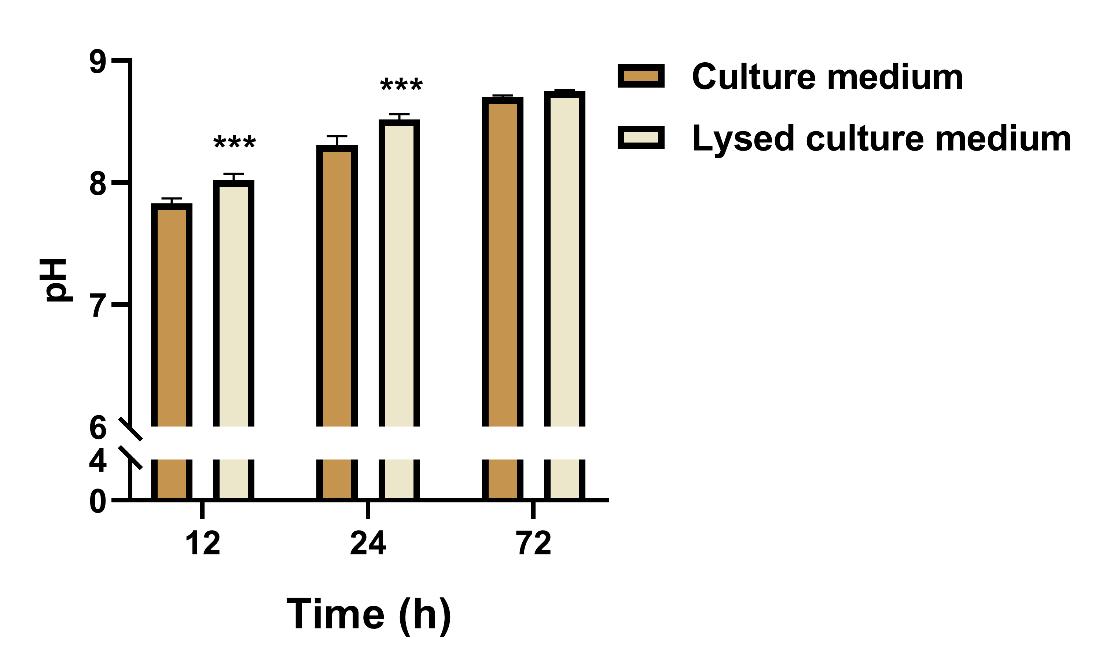


**Figure S1. Alterations in pH levels within cell cultures following pressure lysis.** The OR-15 culture was obtained following incubation in R2A medium for 12, 24, and 72 h (28 ℃,150 rpm). Bacterial cells were lysed using a low-temperature ultra-high-pressure continuous flow cytometer (JN-MiniPro). The pH of the bacterial suspension was measured both prior and following cell lysis.


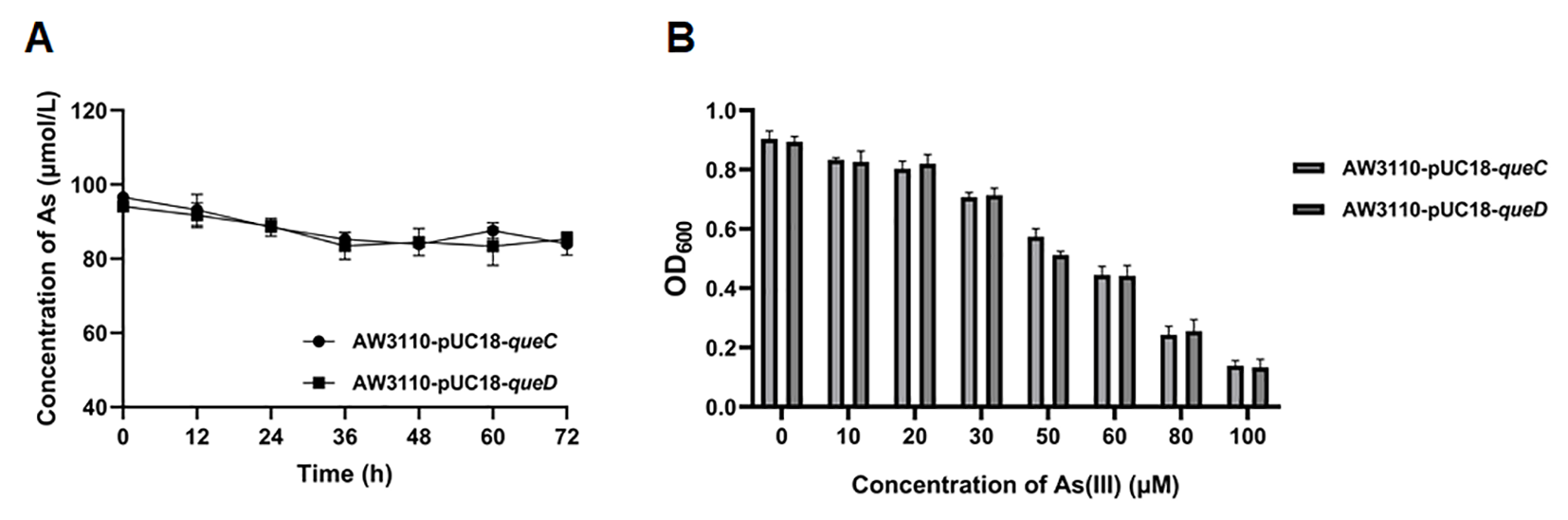


**Figure S2. Phenotype characterization of *queC, queD* heterologous expression strains.** (A) As(III) removal curve of the *queC* or *queD* heterologous expression strains AW3110 at a concentration of 0.1 mM. (B) MIC of As(Ⅲ) for the *queC* or *queD* heterologous expression strains. These data represent average values obtained from three biological replicates.


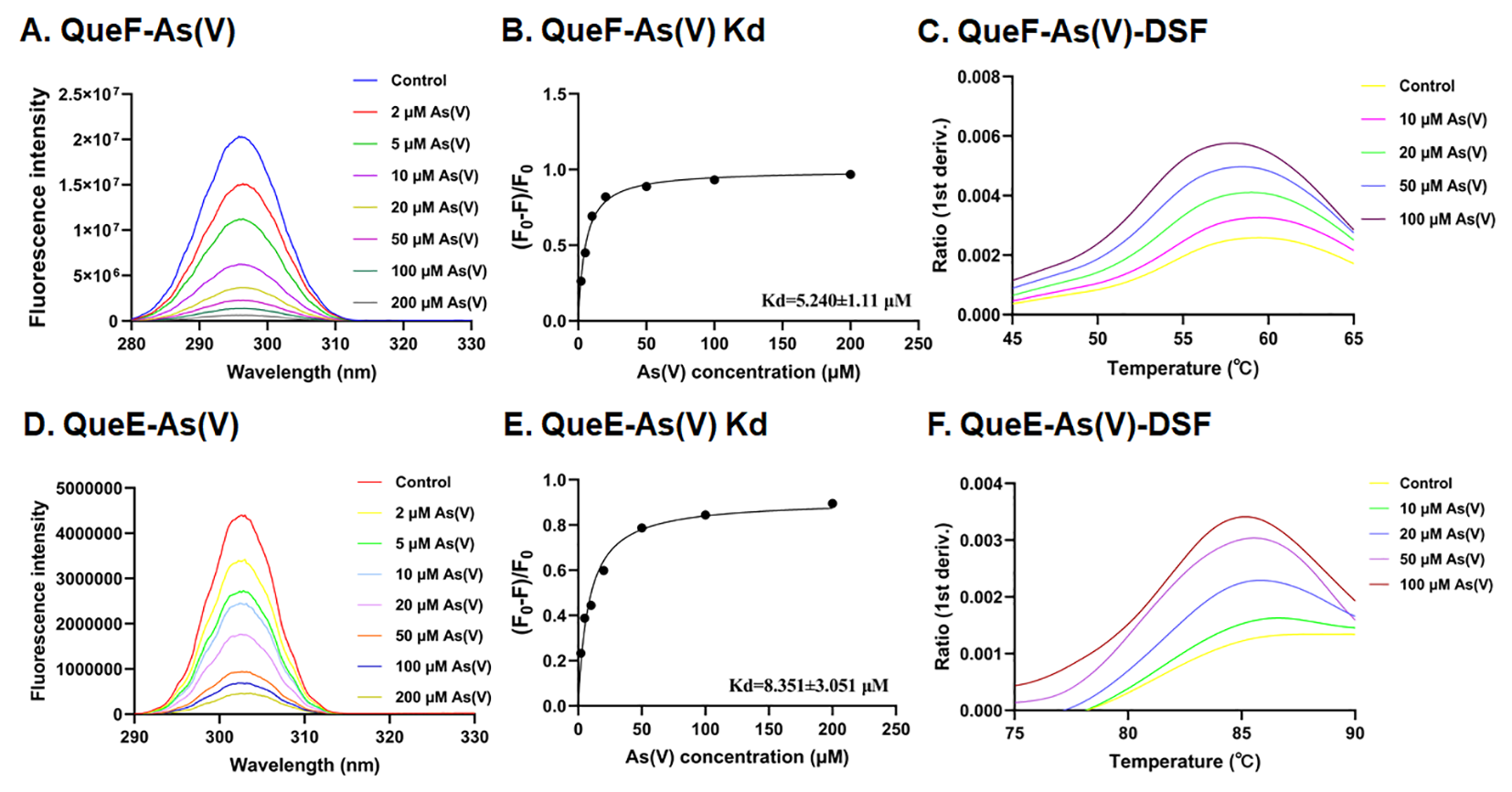


**Figure S3. Interactions of As(V) with QueF and QueE.** (A) Fluorescence quenching was observed upon incubation of purified QueF protein with varying concentration of As(V) (0, 2, 5, 10, 20, 50, 100, and 200 μM). (B) The Kd value for the binding of QueF to As(V) was calculated from the fluorescence decay data. (C) The binding affinity of QueF protein to As(V) at concentrations of 0, 10, 20, 50, and 100 mM was observed by nanoDSF. (D) Fluorescence quenching was observed when purified QueE protein was incubated with the same concentrations of As(V). (E) The Kd value for the binding of QueE to As(V) was calculated from the fluorescence decay data. (F) The binding of QueE protein to As(V) at the same concentrations was observed by nanoDSF.


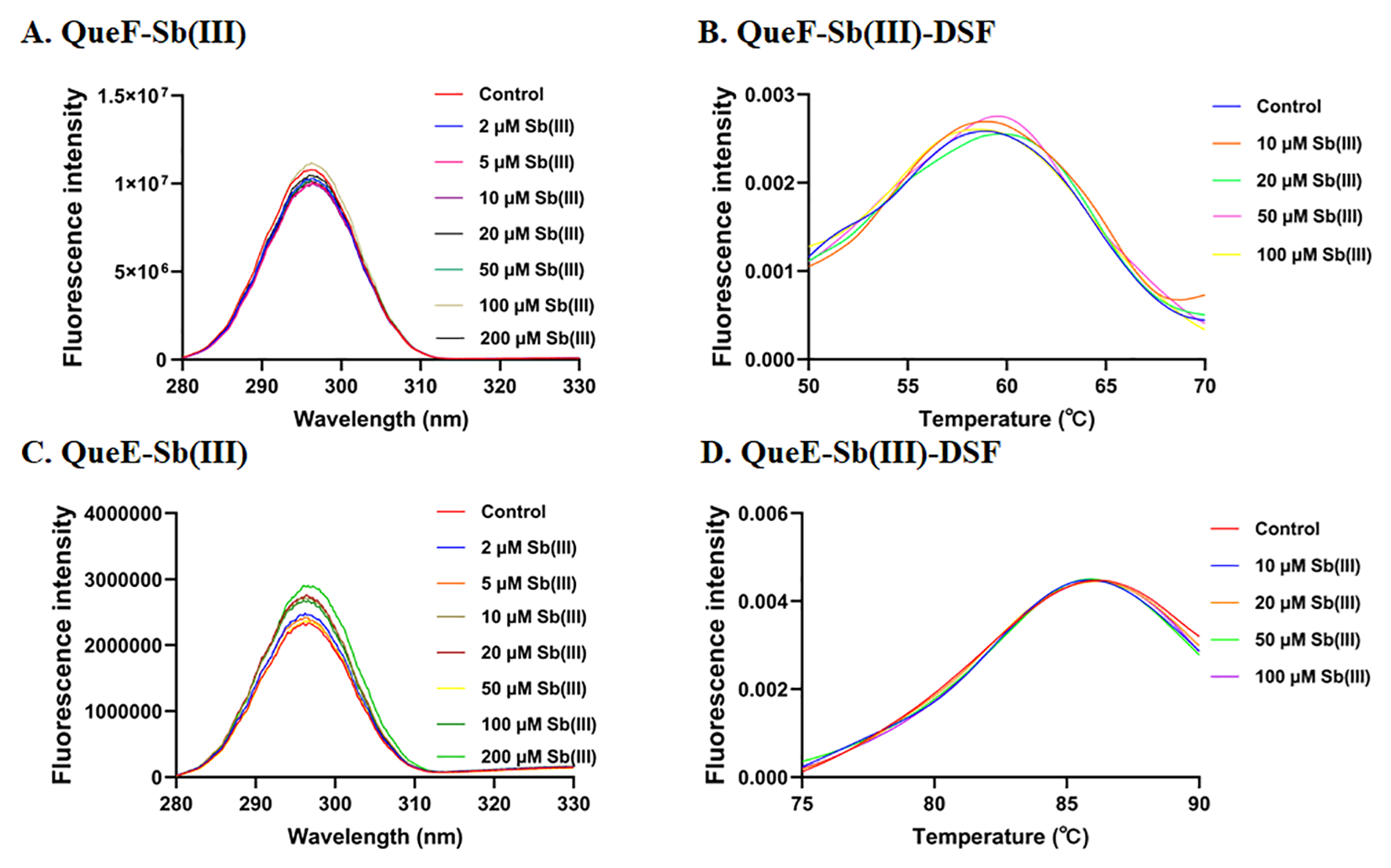


**Figure S4. Fluorescence quenching and nanoDSF detection of Sb(III) with QueF and QueE.** (A) Fluorescence quenching was observed upon incubation of purified QueF protein with varying concentrations of Sb(III) (0, 2, 5, 10, 20, 50, 100, and 200 mM). (B) NanoDSF assays were conducted on QueF protein incubated Sb(III) at concentrations of 0, 10, 20, 50, and 100 mM. (C) Fluorescence quenching was observed when purified QueE protein was incubated with the same concentrations of Sb(III). (D) NanoDSF assays were performed on QueE incubated with the same concentrations of Sb(III).


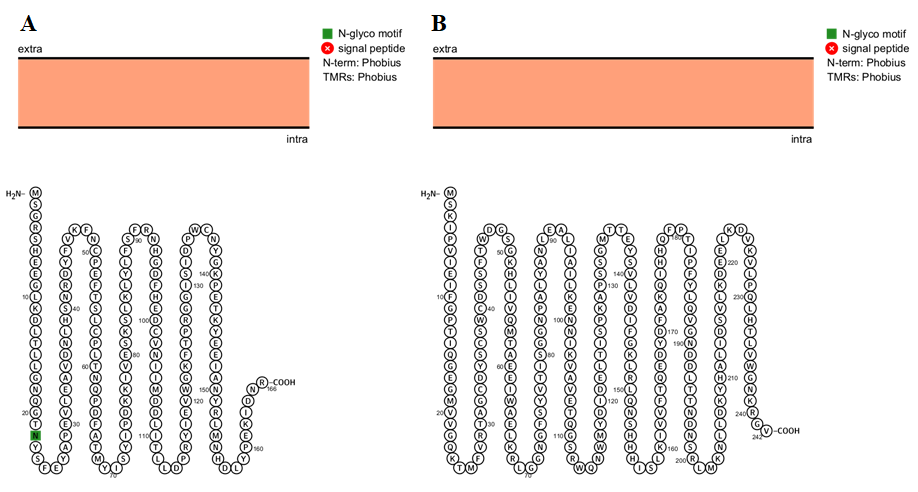


**Figure S5. Prediction of Transmembrane Domains in Proteins.** Prediction of transmembrane domains in the proteins QueF (MBI6864646.1) and QueE (MBI6864643.1) from *Lysinibacillus* sp. OR-15 (A, B) The transmembrane properties of QueF and QueE proteins were predicted using the online tool PROTTER.


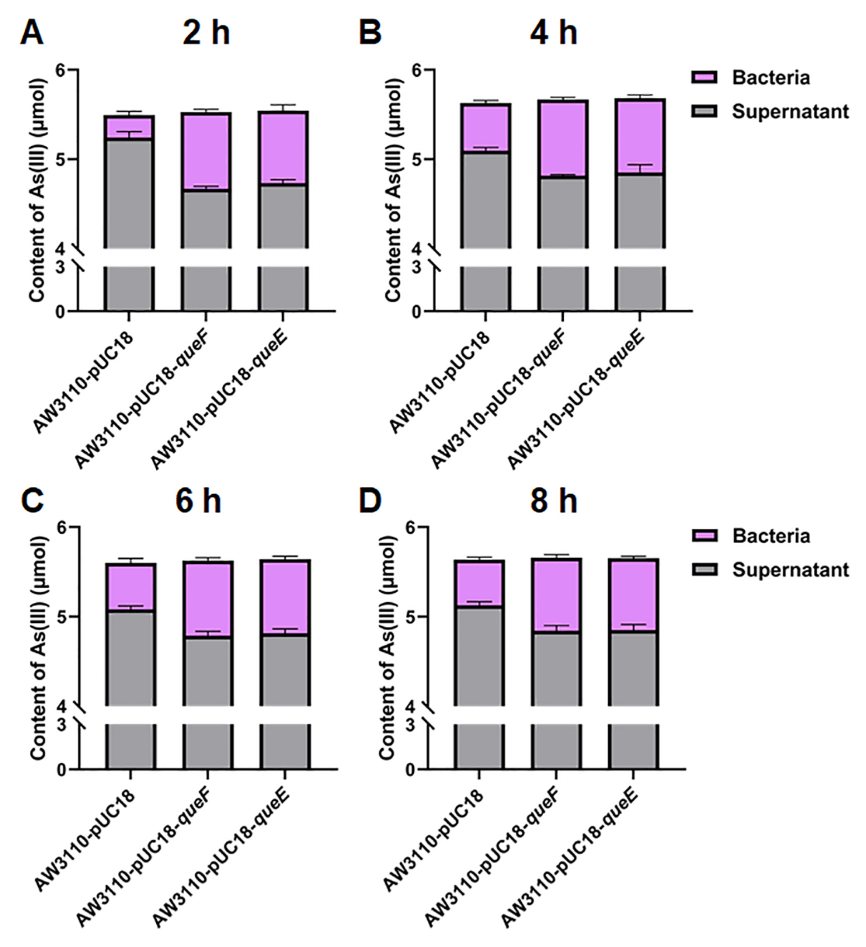


**Figure S6. Detection of total arsenic in resting cell exposure experiments.** Arsenic levels in the supernatant and bacterial biomass after 2, 4, 6, and 8 h of arsenic exposure.


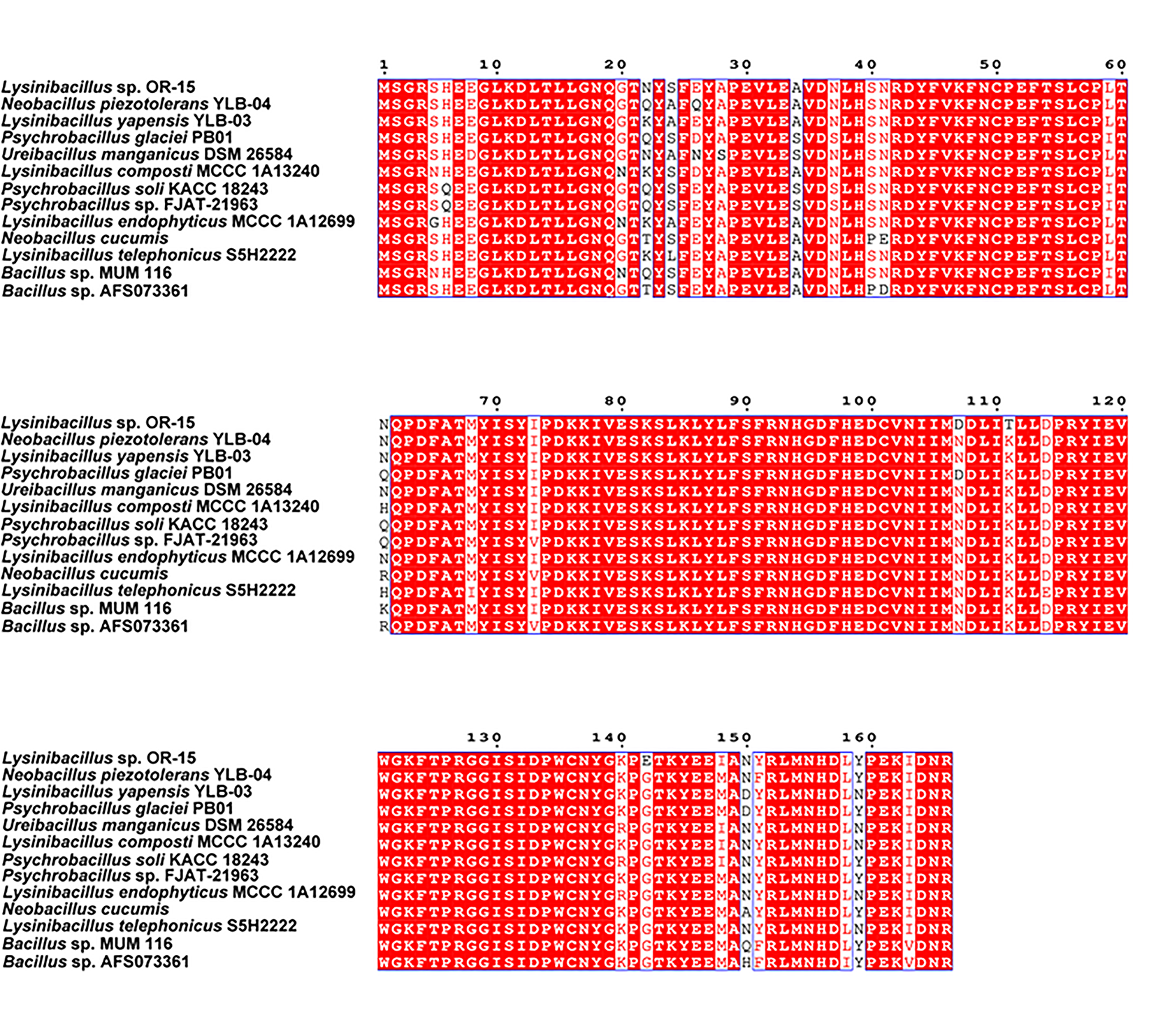


**Figure S7. Multiple sequence alignment of QueF orthologs.** The protein sequence of QueF from *Lysinibacillus* sp. OR-15 (MBI6864646.1) was aligned to QueF ortholog sequences from *Neobacillus piezotolerans* YLB-04 (RDU37369.1), *Lysinibacillus yapensis* YLB-03 (RHW35867.1), *Psychrobacillus glaciei* PB01 (QFF97593.1), *Ureibacillus manganicus* DSM 26584 (KGR77597.1), *Lysinibacillus composti* MCCC 1A13240 (RQW72330.1), *Psychrobacillus soli* KACC18243 (TQR16502.1), *Psychrobacillus* sp. FJAT-21963 (KQL37363.1), *Lysinibacillus endophyticus* MCCC 1A12699 (RKQ15320.1), *Neobacillus cucumis* (PLS05556.1), *Lysinibacillus telephonicus* S5H2222 (RTQ89696.1), *Bacillus* sp. MUM 116 (OIK11280.1), *Bacillus* sp. AFS073361 (PFP26587.1). The multiple alignment was performed using Clustal Omega and ESPript 3.0.

**
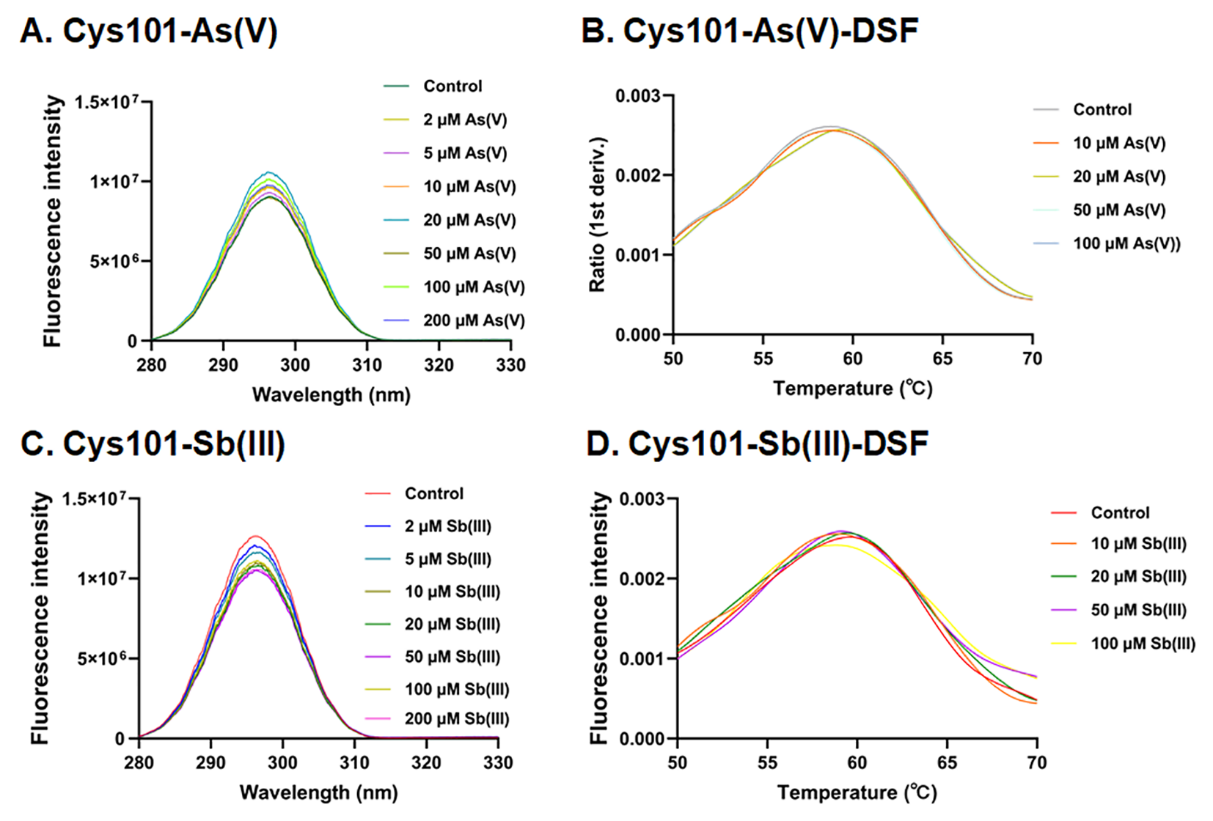
**

**Figure S8. Phenotype of As(V) and Sb(III) binding with Cys101 protein.** (A) Fluorescence quenching was observed upon incubation of purified Cys101 protein with varying concentrations of As(V) (0, 2, 5, 10, 20, 50, 100, and 200 mM). (B) NanoDSF assay was conducted on Cys101 protein incubated with 0, 10, 20, 50, and 100 mM As(V). (C) Fluorescence quenching was observed when purified Cys101 protein was incubated with the same concentrations of Sb(III). (D) NanoDSF assay of Cys101 protein incubated with the same concentrations of Sb(III).


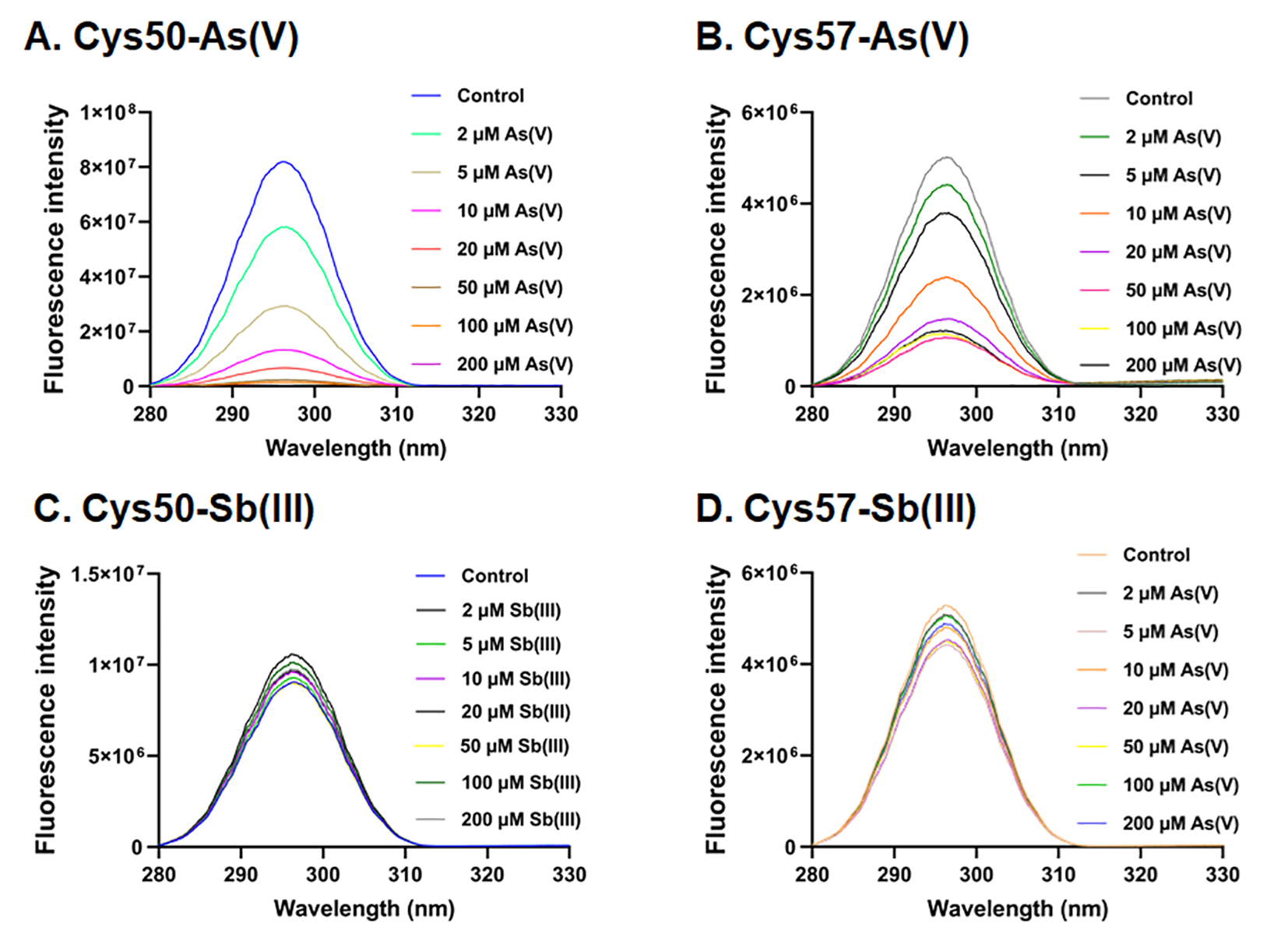


**Figure S9. Interactions of point mutant QueF proteins with As(V) and Sb(III).** Fluorescence quenching was observed for the Cys50 (A) and Cys57 (B) point mutant proteins upon incubation with varying concentrations of As(V) (0, 2, 5, 10, 20, 50, 100, and 200 μM). Fluorescence quenching was observed for the Cys50 (C) and Cys57 (D) point mutant proteins when incubated with the same concentrations of Sb(III).


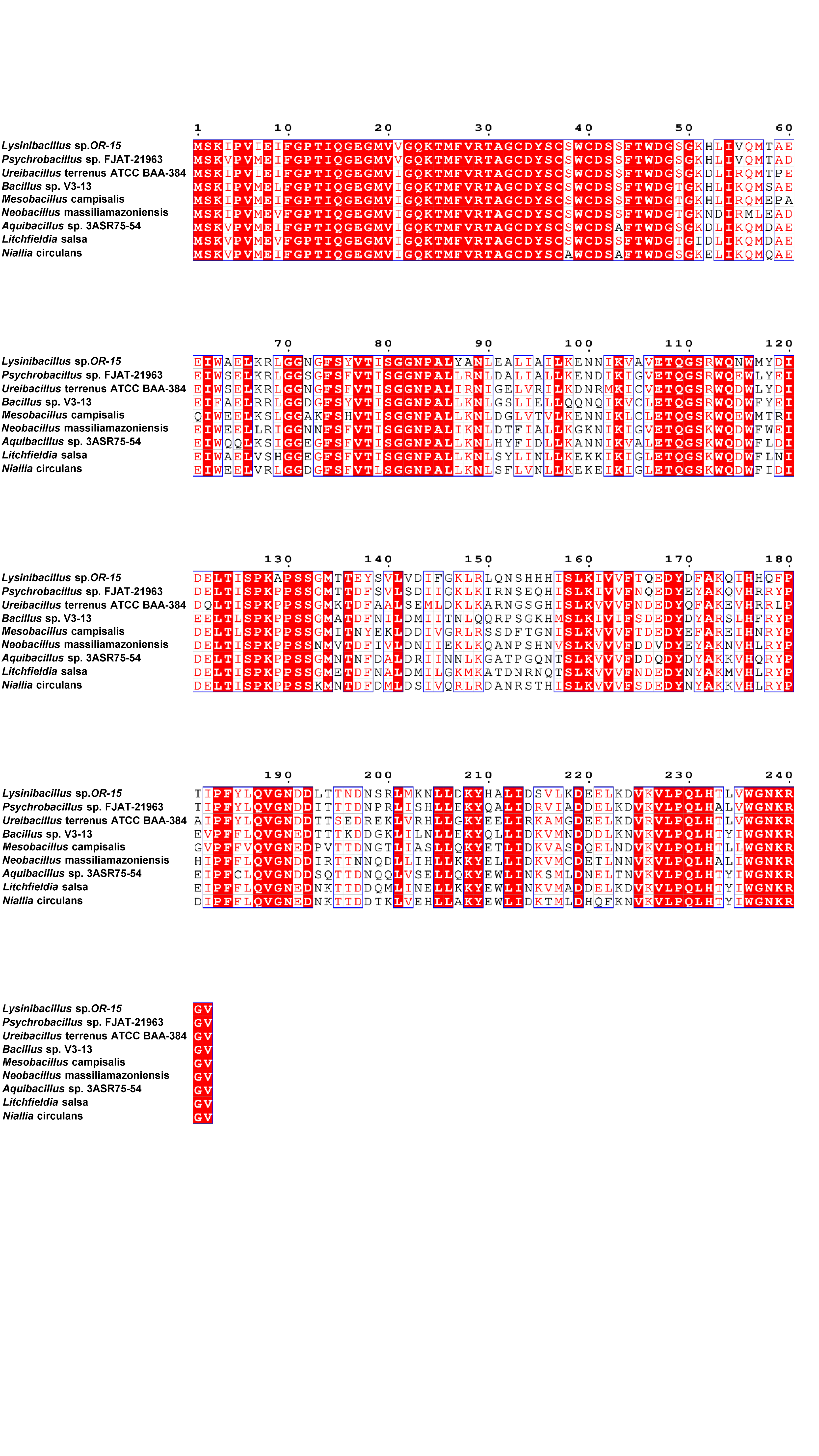


**Figure S10. Multiple sequence alignment of QueE orthologs.** The protein sequence of QueE from *Lysinibacillus* sp. OR-15 (MBI6864643.1) was aligned to QueE ortholog sequences from *Psychrobacillus* sp. FJAT-21963 (KQL37360.1), *Ureibacillus* terrenus ATCC BAA-384 (TQE89040.1), *Bacillus* sp. V3-13 (PLR76565.1), *Mesobacillus* campisalis (KKK38459.1), *Neobacillus* massiliamazoniensis (CRK85109.1), *Aquibacillus* sp. 3ASR75-54 (WP_272447649.1), *Litchfieldia* salsa (SDP12356.1), *Niallia* circulans (KLV27006.1). The multiple alignment was performed using Clustal Omega and ESPript 3.0.


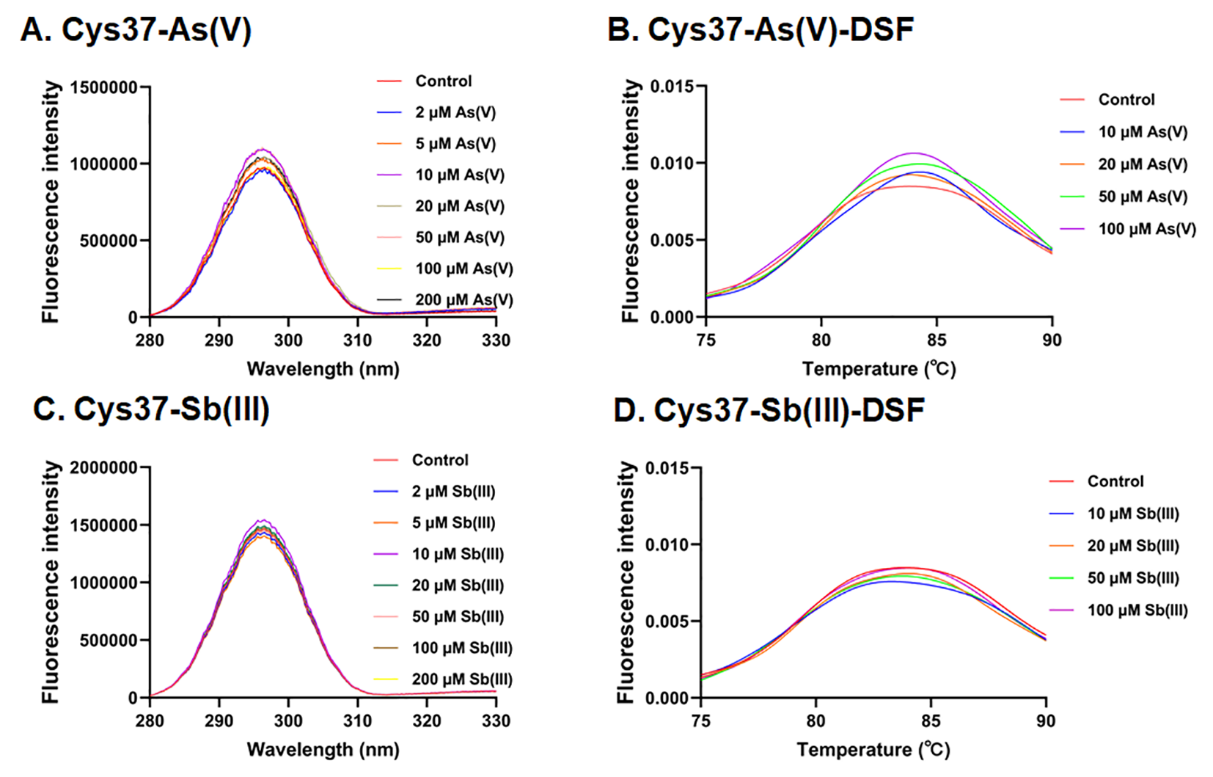


**Figure S11. Phenotypic analysis of QueE Cys37 point mutant protein.** (A) Fluorescence quenching was observed upon incubation of purified Cys37 protein with varying concentrations of As(V) (0, 2, 5, 10, 20, 50, 100, and 200 mM). (B) NanoDSF assay was conducted on Cys37 protein incubated with 0, 10, 20, 50, and 100 mM As(V). (C) Fluorescence quenching was observed when purified Cys37 protein was incubated with the same concentrations of Sb(III). (B) NanoDSF assay was performed on Cys37 protein incubated with the same concentrations of Sb(III).


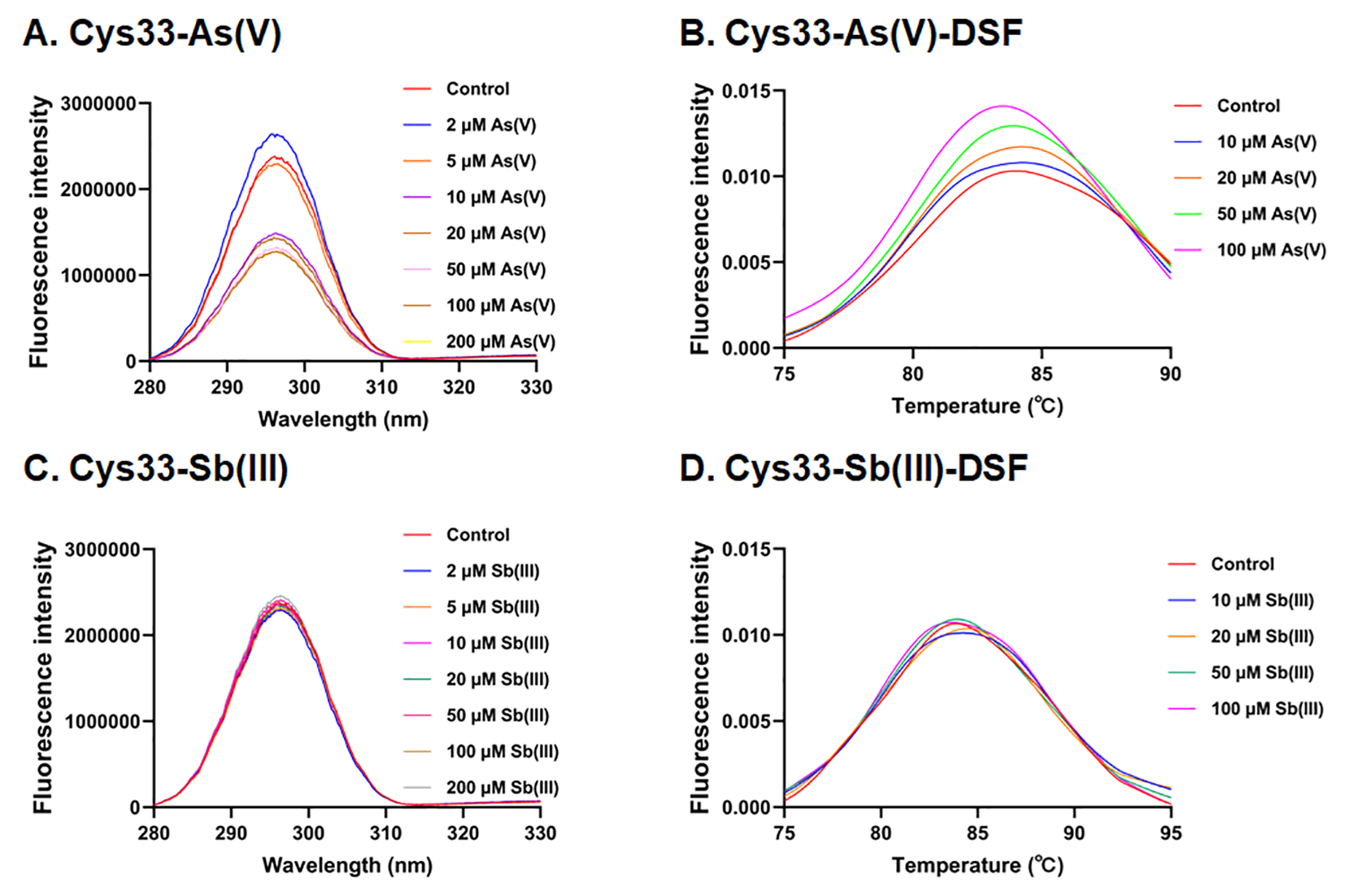


**Figure S12. Phenotypic analysis of QueE Cys33 point mutant protein.** (A) Fluorescence quenching was observed upon incubation of purified Cys33 protein with varying concentrations of As(V) (0, 2, 5, 10, 20, 50, 100, and 200 mM). (B) NanoDSF assay was conducted on Cys33 protein incubated with 0, 10, 20, 50, and 100 mM As(V). (C) Fluorescence quenching was observed when purified Cys33 protein was incubated with the same concentrations of Sb(III). (D) NanoDSF assay was performed on Cys33 protein incubated with the same concentrations of Sb(III).
